# Immunomodulatory effects of intratumoral cowpea mosaic virus and calreticulin nanoparticles in canine tumors: early results

**DOI:** 10.3389/fimmu.2025.1566394

**Published:** 2025-05-02

**Authors:** Akansha Singh, Jessica Fernanda Affonso de Oliveira, Jessica Schrader, Deepan Kishore, Sri Vidhya Chandrasekar, Steven Fiering, Nicole F. Steinmetz, Ashish Ranjan

**Affiliations:** ^1^ Department of Physiological Sciences, College of Veterinary Medicine, Oklahoma State University, Stillwater, OK, United States; ^2^ Aiiso Yufeng Li Family Department of Chemical and Nanoengineering, University of California, San Diego, La Jolla, CA, United States; ^3^ Shu and K.C. Chien and Peter Farrell Collaboratory, University of California, San Diego, La Jolla, CA, United States; ^4^ Center for Nano-ImmunoEngineering, University of California, San Diego, La Jolla, CA, United States; ^5^ Neel Veterinary Hospital, Oklahoma City, OK, United States; ^6^ Department of Microbiology and Immunology, Geisel School of Medicine at Dartmouth, Lebanon, NH, United States; ^7^ Department of Bioengineering, University of California, San Diego, La Jolla, CA, United States; ^8^ Department of Radiology, University of California, San Diego, La Jolla, CA, United States; ^9^ Institute for Materials Discovery and Design, University of California, San Diego, La Jolla, CA, United States; ^10^ Moores Cancer Center, University of California, San Diego, La Jolla, CA, United States; ^11^ Center for Engineering in Cancer, Institute of Engineering Medicine, University of California, San Diego, La Jolla, CA, United States

**Keywords:** cowpea mosaic virus (CPMV) nanoparticles, calreticulin nanoparticle, intratumoral immunotherapy, melanoma, sarcoma, immunomodulation

## Abstract

**Introduction:**

Intratumoral immunotherapy delivers immune-modifying therapeutic agents directly into the tumor microenvironment (TME), stimulating both local and systemic immune responses. In this pilot study, we evaluated the immunomodulatory effects of cowpea mosaic virus (CPMV) particles, which primarily activates innate immunity, and calreticulin nanoparticle (CRT-NP), which enhance immunostimulatory signals of immunogenic cell death in canine cancers. The study focused on their potential to induce local and systemic antitumor immune responses and achieve tumor control.

**Method:**

CPMV was obtained through the mechanical inoculation of Vigna unguiculata, while CRT-NP was generated using cationic liposomes loaded with a CRT-expressing plasmid. Nine canine patients with oral melanoma, soft-tissue sarcoma (STS), and mammary gland carcinoma received CPMV or CRT-NP via intratumoral route. CPMV and CRT-NP was administered weekly at three-five intratumoral locations. To evaluate antitumor immune responses, biopsies and blood samples were obtained prior to treatment and during follow-up visits, extending up to one week after the final treatment. Endpoints included serum cytokine analysis, tumor transcriptomics via NanoString, immune cell profiling in blood and tumor biopsies, and efficacy assessment using RECIST criteria.

**Result:**

CPMV exhibited an icosahedral shape (~30 nm), while CRT-NP were spherical (~300 nm). CPMV induced stable disease (SD) in two of three melanoma and STS patients, with partial response (PR) in the third. CRT-NP induced SD in two of three patients, with one STS patient achieving partial remission. Post-treatment NanoString and flow cytometry analyses revealed a shift in the tumor microenvironment toward a more immunostimulatory state, marked by increased neutrophils and CD8+ T cells. CPMV, in particular, upregulated genes involved in antigen processing and immune activation while enhancing IFNγ+ CD4+ and CD8+ T cell populations. CRT-NP reduced Tregs in the TME. Further, serum cytokine levels, such as MCP-1, GM-CSF, IL-2, IL-6, IL-7 and IL-18, correlated with tumor growth independent of various treatments.

**Discussion:**

Our findings suggest that CPMV and CRT-NP, which activate distinct immunologic pathways, safely modulate the TME contributing to disease stabilization in spontaneous canine cancers. These results support the need for larger-scale trials to address current limitations, differentiate tumor-agnostic versus treatment-specific effects, and evaluate long-term clinical outcomes, including overall survival (OS).

## Graphical Abstract

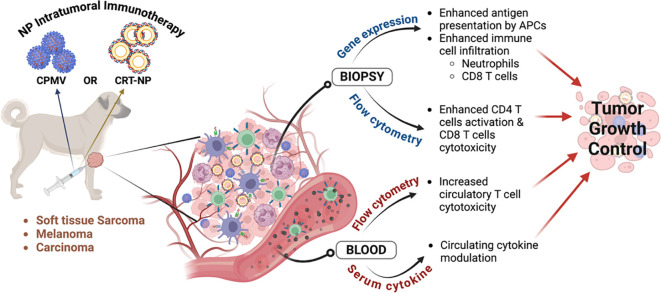

## 1 Introduction

Intratumoral immunotherapy directly administer immune-modifying therapeutic agents such as immune checkpoint inhibitors, cytokines, or oncolytic viruses into the tumor microenvironment, thereby stimulating both local and systemic immunity against the cancer cells (1). Multiple preclinical studies in murine models have shown that intratumoral therapy significantly boost antitumor efficacy by activating both innate and adaptive immune systems (2, 3). Clinical trials further support these findings, with intratumoral injections of checkpoint inhibitors like pembrolizumab and oncolytic viruses such as talimogene laherparepvec (T-VEC) demonstrating improved response rates and overall survival in patients with melanoma and other cancers (4). Building on this concept, we and others are focusing on developing intratumoral therapeutic nanoparticles (NPs) for local and systemic immune-modulation against solid tumors. Particularly, we have shown that intratumoral administration of cowpea mosaic virus (CPMV) particles evokes strong antitumor immunity by activating both innate and adaptive immune responses across various murine tumor models. CPMV stimulates a potent antitumor immune response in mouse models of melanoma, ovarian cancer, breast cancer, colon cancer (5, 6), and glioma (7). CPMV does not replicate in mammals and can be administered in high doses with minimal toxicity. CPMV and its RNA-free counterpart, “empty (e) CPMV”, are highly immunogenic, efficiently produced in plants, scalable to produce, and chemically modifiable (8, 9). CPMV injected directly into an identified primary tumor relieve immunosuppression by inducing fast recruitment of antigen presenting cells (APCs) in tumors (7). Innate cells in tumors tend to be immunosuppressive and have poor antigen-presenting capabilities; CPMV treatment changes the tumor microenvironment (TME) to immunostimulatory, i.e., reprogramming M2 macrophages to M1 macrophages and infiltrating N1 neutrophils. These innate immune cells have activated receptors [TLRs; specifically, CPMV activates TLR2, 4, and 7 (10)] and mature, process antigens within the tumor, and express CD80, CD86, and MHC Class II. The innate cells then travel to the draining lymph nodes, where they effectively present the tumor-associated antigens. This cascade of events leads to adaptive responses, including increased tumor infiltration by tumor antigen-specific CD4+ and CD8+ effector T cells and memory T cells. CPMV has demonstrated efficacy as solo-treatment as well as in combination with radiation (11), chemotherapy (12), and checkpoint inhibitors (13), amongst others.

Similarly, we have demonstrated that delivering a calreticulin (CRT) expression plasmid via a cationic liposome nanoparticle (CRT-NP) generates an immunostimulatory signal in mice tumors, akin to the immunogenic cell death-associated external cell membrane-bound CRT induced by radiation (14–16). CRT is a well-known multifunctional chaperone protein involved in cellular calcium homeostasis and immune modulation. Our exogenous delivery approach effectively reduced the volumes of both treated (injected) and contralateral untreated tumors. As we continue to refine these treatments in murine models, understanding the immune dynamics of these NPs in clinically relevant patient populations is crucial.

Canine cancers develop naturally and closely resemble human malignancies in histological features, genetic alterations, and TME characteristics, including immune characteristics (17, 18). Unlike genetically engineered mouse models, which may not fully replicate human cancer complexity, canine cancers provide a more accurate representation of natural disease progression and therapeutic response since they are spontaneous tumors in large, generally older animals, like in human patients. The similarities between canine and human cancers extend to genetic and molecular pathways. For instance, mutations in genes such as BRAF and NRAS are common in both canine and human melanomas, making canine melanoma a valuable model for studying melanoma-specific immunotherapies (19). Canine osteosarcoma like human osteosarcoma exhibit comparable patterns of bone remodeling and genetic alterations, such as mutations in the p53 tumor suppressor gene. The TME in canine osteosarcoma, including the interactions between osteoblastic cells and the surrounding stroma provides an accurate model for studying novel immunotherapeutic approaches targeting bone tumors (20). Although the activation of immune cells like T cells and macrophages varies in functional profiles (e.g., lower IFNγ production by canine T-cells compared to humans (18), clinical trials in canine patients with spontaneous tumors still have the potential to bridge the gap between mouse models and human patients.

The primary aims of this study were to evaluate the ability of intratumorally administered CPMV and CRT-NP to achieve local tumor control and assess the associated antitumor immune responses in canine cancer patients. The NPs were selected for their distinct immunostimulatory mechanisms and because they showed promising efficacy in murine xenograft models. In terms of physicochemical characteristics, CPMV NP is a plant-derived, replication-incompetent product that attracts innate immune cells to the TME. In contrast, CRT-NP is synthesized using lipids and a plasmid designed to transfect tumor cells and induce CRT overexpression. We hypothesized that despite their distinct mechanisms of action, both would induce tumor growth control, targeting different pathways of antitumor immunity. Given the novelty of our NPs in canine oncology, a small cohort was chosen to assess therapeutic safety as an initial step to confirm whether the immunomodulatory effects observed in mice translate to spontaneous canine tumors, justifying resource mobilization for future efficacy and survival studies in canine and potentially human trials. Data suggest that intratumoral immunotherapy using nanoparticles induces local immunomodulation and can stabilize cancer progression in some cases.

## 2 Materials and methods

### 2.1 Synthesis of CPMV and CRT-NP

CPMV was obtained by the mechanical inoculation of (*Vigna unguiculata*, California Black Eye from Mountain Valley Seed, Lot 175027) followed by purification and characterization as previously described (21). Then, a NanoDrop 2000 spectrophotometer (Thermo Fisher Scientific) was used to measure CPMV concentration using the Beer-Lambert law (ϵCPMV at 260 nm = 8.1 mL.mg-1.cm-1). Particle integrity was analyzed by size exclusion chromatography (SEC) using the Äkta Go FPLC fast protein liquid chromatography system (Cytiva) fitted with a Superose 6 Increase 10/300 GL column operating at a flow rate of 0.5 mL/min. CPMV was eluted (0.5 mg/mL using 0.1 M potassium phosphate (KP) buffer (pH 7.0) as the mobile phase) using an isocratic elution profile with absorbance detector fixed at 260 nm (nucleic acid). Denaturing SDS-PAGE was used to evaluate CPMV coat proteins. Briefly, CPMV (10 µg) was mixed with 5 µL of 4x lithium dodecylsulfate (LDS) sample buffer (Life Technologies) and 1.6 µL of 10x NuPAGE sample reducing agent (Invitrogen), followed by denaturation at 95°C for 5 minutes. SeeBlue™ Plus2 Pre-stained Protein Standard (8 µL) and denatured CPMV were loaded onto a precast NuPAGE 4–12% Bis-Tris Protein Gel and proteins were separated at 200 V for 40 min using 1x NuPAGE MOPS running buffer (Invitrogen). Gel was imaged under white light to visualize the protein after staining with GelCode Blue Safe Protein Stain (Thermo Fisher Scientific). A Jeol 1400Plus (Peabody) transmission electron microscope (TEM) was used to obtain images from CPMV nanoparticles. Briefly, CPMV (0.1 mg/mL in 20 µL of deionized water) was loaded onto a glow-discharged (PELCO easiGlow) 400-mesh hexagonal copper grids (Electron Microscopy Sciences). After being washed twice with deionized water (20 µL), sample was negatively stained with 2% (w/v) uranyl acetate (Electron Microscopy Sciences) and sample was imaged at 80,000x magnification at 80 kV. Endotoxin quantification was performed using Pierce Chromogenic Endotoxin Quant Kit (ThermoFisher Scientific), where clinical acceptable CPMV endotoxin limit was calculated as 35.7 EU/mg, based on the estimated efficacious CPMV dosage of 10 mg/tumor in humans and following the FDA guidance for industry, to convert the dose between humans and mice (22). We considered an average human body weight of 70 kg for this calculation.

For CRT-NP synthesis, we inserted the human CRT gene into an antimicrobial resistance (AMR) gene-free NTC plasmid backbone (Nature Technology Corp., NE), an ideal non-viral vector for translation to human trials *vs*. pCMV3 expression systems with AMR genes. CRT is a highly-conserved 46 kDa endoplasmic reticulum (ER) protein, with conserved amino acid sequence homologies between mice and humans (23). CRT gene cloned in NTC plasmid was loaded in cationic liposomes to synthesize CRT-NP as follows. A lipid film was hydrated in 10 mM HEPES buffer (pH 7.4) at 55°C, and the lipid suspension was then extruded five times through filters of 200 nm pore size to yield homogeneous liposome (24). Plasmid were added to the DNA solution in the liposomes vial (20:1; lipid:plasmid; wt:wt), gently mixing it by pipetting. NPs were incubated at room temperature for 30 min and then stored at 4°C until used. We assessed the size (hydrodynamic diameter), polydispersity index, and zeta (z)-potential of the resultant CRT-NP stored at 4°C using dynamic light scattering (DLS) with a 90-plus PALS Nanobrook device. Transmission electron microscopy (JEOL JEM-2100, Peabody, MA, USA) was conducted to evaluate the morphology and stability of CRT-NPs stored for up to 3 days.

EMBL-EBI multiple sequence alignment tool, Clustal Omega, was used to establish percent identity between human (Accession: AAB51176.1) and dog (Accession: XP_038284378.1) CRT protein. Google Deepmind and Isomorphic labs structure prediction tool, AlphaFold3 was used to predict dog CRT protein using protein sequence from NIH GenPept (Accession: XP_038284378.1). Human CRT protein structure was used from EMBL-EBI-AlphaFold database.

We tested cytotoxicity of CRT-NP against two canine cancer cells, OSCA78 osteosarcoma cell line (#EMN004-FP, Kerafast, MA, USA) and CMGD5 melanoma cell line (#EMN007-FP, Kerafast, MA, USA). Both cell lines were maintained in DMEM media (#11965092, Gibco, Thermofisher, CA, USA) with 10% FBS (#F4135, MilliporeSigma, MA, USA), 50 U/ml Pen-Strep solution (#15070063, Gibco, Thermofisher, CA, USA) and 10mM HEPES (#15630106, Gibco, Thermofisher, CA, USA). 50,000 cells were plated in 96 well plate and transfected with CRT-NP, CRT plasmid + LipoFectMax (1:3 ratio; #FP310, ABP Biosciences, MD, USA), and Blank NP (Lipoplex with NTC plasmid without CRT gene) in serum free media. After 6h, media was replaced with complete DMEM media and 48h later MTT assay was done. For extracellular expression of CRT protein on canine cell line, OSCA78and CMGD5 were transfected as mentioned above with 1μg/ml CRT plasmid each well and incubated for 48h before staining with CRT antibody. Cells were washed with PBS and incubated with CRT primary antibody (#PA5-25922, Invitrogen, CA, USA) for 60mins on ice followed by washing with FACS buffer (PBS+2% FBS). Cells were then incubated with FITC conjugated secondary antibody (#31635, Invitrogen, CA, USA) for 60mins on ice in dark. Cells were washed and stained Live-Dead dye following manufacturer’s protocol. Cells were washed thrice and data was acquired using Agilent Novocyte3000 and analyzed using FlowJo software. For western blot analysis, cells treated with similarly with 1μg/ml CRT plasmid as above and stained using primary CRT antibody (#PA5-25922, Invitrogen, CA, USA) and GAPDH (#AM4300, Invitrogen, CA, USA) followed by HRP conjugated secondary antibody (Goat anti-Rabbit #32260, Goat anti-mouse #31430, Invitrogen, CA, USA) incubation. ImageJ was used to quantify band intensities and densitometry graph was generated using Graphpad Prism software v10.3.0.

### 2.2 Pilot canine trial study design and endpoints

**Figure 1:**
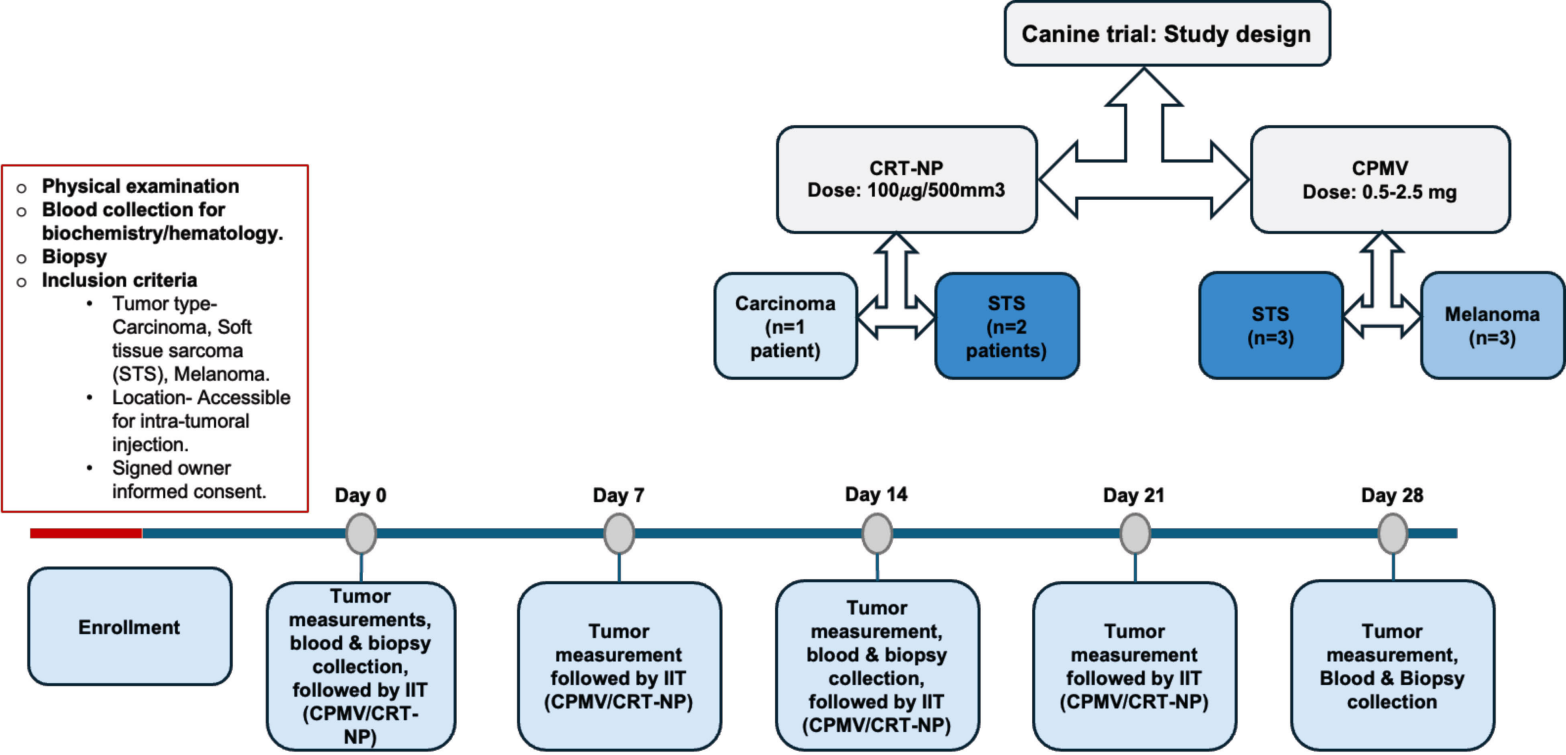
Patients with soft tissue sarcoma (STS), melanoma, or carcinoma with accessible solid tumors for intratumoral immunotherapy (IIT) received four weekly treatments of CPMV or CRT-NP. CPMV was administered at doses ranging from 0.5 to 2.5 mg (0.5 mg for the first two patients, followed by a higher dose for the third). CRT-NP was given at a fixed dose of 100 µg CRT-NP per 500 mm³ of tumor. Tumor dimensions (length and width) were measured weekly, and blood and biopsy samples were collected on the 14th and 28th days after the first treatment for transcriptomic, immune cell and cytokine analysis.

All animal-related procedures were approved by the Oklahoma State University Animal Care and Use Committee (Protocol#21-41). Prior to treatment, owner consent was obtained, including a release from institutional and personal (researcher) liability. Nine privately owned canine patients, aged between 8-13 years, with tumors ranging from 1.8-600 cm³, were enrolled in the study (**Figure 1**). For CPMV treatment, cancer patients (n=3 per group for soft tissue sarcoma (STS) and melanoma) received 0.5-2.5 mg CPMV over four weekly doses. To align with future first-in-human trials, the initial two dogs in both STS and melanoma group received 0.5 mg CPMV each. Since no adverse effects were observed, subsequent dogs received higher doses (1mg and 2.5mg). For CRT-NP, three patients (two with STS and one with carcinoma) received four weekly intratumoral doses of 100 μg CRT-NP per 500 mm³, injected into three-five intratumoral locations. One STS patient did not complete all CRT-NP doses due to the owner’s decision to euthanize the patient by week 2.

Biopsies and blood samples were collected before treatment and during follow-up visits up to 1 week post last treatment to assess antitumor immune responses. Biopsies were collected closer to treatment region with a 6mm Baker’s punch after cleaning the tumor with 2% chlorhexidine solution. Only the largest accessible tumor was treated and evaluated for tumor control. Treatment efficacy was assessed through tumor caliper measurements and the documentation of new lesions, following the Veterinary Cooperative Oncology Group Response Evaluation Criteria in Solid Tumors (RECIST v1.1) guidelines. A complete response was defined as the disappearance of all target lesions. A partial response was characterized by a greater than 30% decrease in the longest diameter of the tumor, while stable disease was noted as a decrease of less than 30% or an increase of less than 20% in the longest diameter of target lesions following nanoparticle treatments. Progressive disease was indicated by the appearance of one or more new lesions or an increase of at least 20% in the longest diameter of target lesions.

### 2.3 Transcriptomic profiling of tumors using NanoString nCounter

#### 2.3.1 RNA extraction and sample preparation for nCounter analysis

For transcriptomic analysis, we examined tumors from seven patients: three STS patients and three melanoma patients treated with CPMV, and one carcinoma patient treated with CRT-NP. Biopsy samples were collected at three time points: pre-treatment (D0), 14 days post-first treatment (D14), and 28 days post-first treatment (D28). Only one sample was available from the CRT-NP-treated patient due to early euthanasia and procedural limitations. For comprehensive multiplex RNA-based immune profiling across various cancer types, total RNA was isolated from CRT-NP and CPMV tumor samples collected in RNAlater solution (n = 3 per group for CPMV and n = 1 for the CRT-NP treated carcinoma bearing patient) using the Trizol extraction method. Briefly, tumors were homogenized in 1 ml of Trizol reagent and incubated at room temperature (RT) for 5 minutes. Following this, 200 μl of chloroform was added, and the samples were vortexed and incubated for an additional 10 minutes at RT. The samples were then centrifuged at 12,000 RPM for 15 minutes at 4°C (Microfuge 5418, Eppendorf), and the clear aqueous phase containing RNA was carefully collected into a fresh tube. RNA was precipitated using isopropanol, and the RNA pellet was separated by centrifugation at 12,000 RPM for 10 minutes at 4°C. The pellet was washed with 75% ethanol, resuspended in RNase-free water, and treated with DNase to remove any residual DNA contamination.

Sample concentration was adjusted to 5 ng/μl for nCounter analysis using the Canine IO 800 gene panel (NanoString Technologies, Seattle, WA, USA) according to the manufacturer’s protocol. Briefly, 70 μL of hybridization buffer was added to the reporter CodeSet and mixed thoroughly to prepare the master mix. A volume of 8 μL of the master mix was added to 5 μL of RNA samples in NanoString-provided strip tubes. The samples were mixed thoroughly by inversion, briefly centrifuged, and then set for hybridization at 65°C for 16 hours. After hybridization, samples were washed to remove excess probes, and the biotinylated hybrids were immobilized on a streptavidin-coated cartridge using the NanoString nCounter Preparation Station (NanoString Technologies). The nCounter Digital Analyzer (NanoString Technologies) was then used to count barcodes and quantify the target genes in each sample.

#### 2.3.2 Gene count analysis

Raw data was normalized and log-transformed using nSolver version 4.0 (NanoString Technologies). Normalization was performed using the geometric mean of positive controls and housekeeping genes. Samples were annotated based on time and analyzed using the Advanced Analysis 2.0 plugin of the nSolver software for differential expression (DE) of genes, with pre-treatment biopsy samples (D0) serving as the baseline and day 14 (D14) and day 28 (D28) samples as categorical covariates. The Benjamini-Hochberg method was employed to estimate the false discovery rate (FDR) for p-value adjustment.

Gene counts were further analyzed using the online tool BigOmicsAnalytics (https://bigomics.ch) to generate immune cell profiling plots and geneset UMAPs. For immune cell profiling, the Non-Negative Least-Squares (I-NNLS) test method was applied through the Immune Cell (LM22) database to identify and score immune cell signatures and generate dot plots. UMAPs were colored by relative log expression, using the covariance distance metric. Clustered foldchange heatmap grouped similar profile genes that were either up/downregulated at both timespans, i.e. D14 or D28 from pretreatment samples. Gene expression heatmap were generated on scaled log-expression values (z-score) using Euclidean distance and Ward linkage. Cytokine and TNF family gene expression heatmap were generated using BigOmincsAnalytics in-built databases. Gene enrichment analysis was done using online GO tool ShinyGO 0.81 (SDSU, USA; http://bioinformatics.sdstate.edu/go/) using Gene Ontology Biological process (BP) database for Dog species. For functional protein enrichment analysis, STRING v12.0, was used.

Raw gene counts were also uploaded to the ROSALIND platform (ROSALIND, San Diego, CA, USA; https://www.rosalind.bio) for DE analysis of CPMV-treated STS and melanoma samples, generating volcano plots with log2 fold change on the x-axis and -log10(p-value) on the y-axis. Data normalization followed the same method as in nSolver, and the Benjamini-Hochberg method was used for p-value adjustment. Genes with a fold change greater than 1.5 and p < 0.05 were considered significant and were used for further analysis.

### 2.4 Immunophenotyping of immune cells in blood and tumor biopsies with flow cytometry

#### 2.4.1 Sample collection and preparation

Blood samples from dogs treated with CPMV and CRT-NP were obtained using BD Vacutainer EDTA tubes. Tumor biopsies were immediately placed in RPMI medium enriched with 2% fetal bovine serum (FBS). To prepare single-cell suspensions, the tumor tissues were first mechanically disrupted, then subjected to enzymatic digestion with 200 U/ml collagenase IV (Life Technologies, Grand Island, NY, USA) at 37°C for one hour. The resulting cell preparations were passed through a 70 µm cell strainer (Corning Inc., Corning, NY, USA). For the blood samples, a 1× red blood cell lysis buffer (multi-species, Invitrogen, Waltham, MA, USA) was used, with incubation lasting 10–15 minutes before proceeding to antibody staining.

#### 2.4.2 Antibody staining

The staining process involved a 60-minute incubation on ice in the dark with the following antibodies: anti-CD3+, anti-CD4+, anti-CD8+ (dog T lymphocyte cocktail, cat. 558699, BD Pharmingen, San Diego, CA, USA). For the detection of IFNγ and Foxp3+ T regulatory (Treg) cells, the procedure continued with washing the cells post surface marker staining. Cells were then fixed, permeabilized using a transcription factor buffer set (BD Biosciences, San Jose, CA, USA), and incubated with e-fluor 450 labeled anti-Foxp3 (#48577382, Invitrogen, CA, USA). Before proceeding with IFNγ staining, cells were incubated to goat anti-mouse unconjugated antibody (#AI-9200-1.5, Vector laboratories, CA, USA) for 60 minutes on ice in the dark to block earlier mouse IgG light and heavy chains. Cells were washed with flow buffer (PBS+ 2% BSA) and incubated with unconjugated mouse anti-IFNγ (#MCA1783, Biorad) for 60 minutes on ice in the dark followed by staining with PE-Cy7 labelled rat anti-mouse secondary antibody (#25401582, Invitrogen, CA, USA) for 15mins on ice in dark. Cells were washed thrice with flow buffer before acquisition on Novocyte3000 (Agilent, Santa Clara, CA, USA). Unstained and single stained cells were used for compensation and gating on NovoExpress software v1.6.2 (Agilent, Santa Clara, CA, USA).

### 2.5 Serum cytokine analysis and their correlation with therapeutic efficacies

Canine cytokines, chemokines, and growth factors were analyzed using the Luminex™ 200 system (Luminex, Austin, TX, USA) at Eve Technologies Corp. (Calgary, Alberta). The analysis utilized the Canine Cytokine 13-Plex Discovery Assay^®^ (MilliporeSigma, Burlington, Massachusetts, USA), which includes the following 13 analytes: GM-CSF, IFNγ, IL-2, IL-6, IL-7, IL-8/CXCL8, IL-10, IL-15, IL-18, IP-10/CXCL10, KC-like, MCP-1/CCL2, and TNFa, with sensitivity ranging from 0.5 to 50,000 pg/mL depending on cytokine. From the pre-treatment serum samples of all patients, GM-CSF, IL-2, IL-6, IL-7, IL-8/CXCL8, IL-10, IL-15, IL-18, KC-like, and MCP-1/CCL2 were reliably detected. These cytokines were then compared with their levels in post-treatment samples. Fold changes in data were calculated using the formula: (post-treatment levels/Pre-treatment levels) - 1 and plotted to differentiate between NP treatment and patient tumor type.

### 2.6 Statistical and correlation analysis

All statistical analyses were conducted using Prism v10.3.0 (GraphPad Software Inc., La Jolla, CA, USA). Changes in immune cell populations and cytokine levels over time were assessed using a paired t-test, assuming normal distribution and equal variance. Group comparisons based on treatment type and tumor groupings were performed using a two-way ANOVA with Fisher’s LSD test, under the assumptions of normality and homogeneity of variances, verified by Shapiro-Wilk and Levene’s tests, respectively. Since all assumptions were met, data transformation was not required. Bivariate Pearson’s correlation (Pearson’s r) was used to evaluate linear relationships between multiple variables, assuming normality and linearity. Log transformation was applied where necessary to address violations of normality. Correlation heatmaps were color-coded to indicate direction (green/blue for positive, red for negative) and strength of correlations.

## 3 Results

### 3.1 CPMV and CRT-NP particle characterization

**Figure 2:**
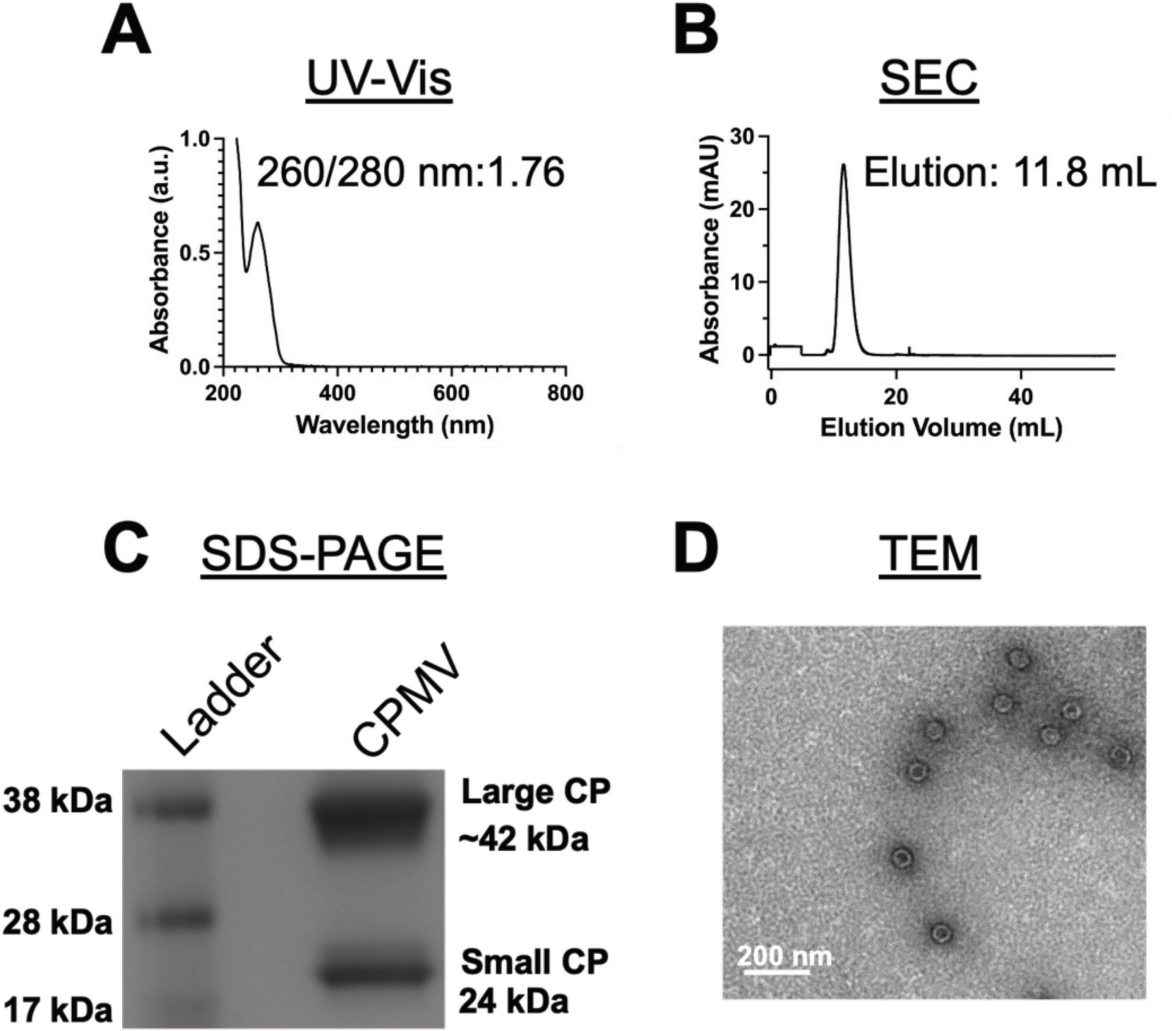
Physicochemical characterization of CPMV. **(A)** UV-Vis spectra of CPMV showing RNA:protein A260/280 ratio of 1.76, indicating sample purity. **(B)** Size exclusion chromatography profile of CPMV from a Superose6 Increase columns showing the typical elution profile and absence of aggregates or broken particles, indicating the integrity of the purified particles. **(C)** CPMV small (24 kDa) and large (42 kDa) coat proteins were visualized after separation on a denaturing 4−12% Nu-PAGE gel, followed by staining GelCode Blue Safe protein stain and imaged under white light. **(D)** Transmission electron microscopy (TEM) images of negatively stained CPMV particles showed their icosahedral shape and confirmed the absence of aggregates. Sample was imaged at 80,000x magnification at 80kV. The image data were analyzed using ImageJ. Graphs in panels A and B were generated using GraphPad Prism v10.2.0 software.

CPMV concentration and RNA:protein ratio was evaluated by UV-Vis (**Figure 2A**), revealing a A260/280 nm ratio of ~1.7, confirming the RNA quality and particle purity (25). Size exclusion chromatography (SEC; **Figure 2B**) shows CPMV characteristic elution profile at ~11.8 mL, and the absence of broken particles or aggregates confirmed the sample purity. SDS-PAGE electrophoresis gel (**Figure 2C**) revealed the presence of both CPMV large (~42 kDa) and small (~24 kDa) coat proteins, while transmission electron microscopy (TEM; **Figure 2D**) image showed the icosahedral shape of these particles and absence of aggregation (the image is representative of multiple analysis). Endotoxin assay was performed to ensure these particles were suitable for clinical usage; with exception of intrathecal route, FDA recognizes 5.0 EU/kg threshold pyrogen dose of endotoxin for administration routes (26). Pierce Chromogenic Endotoxin Quant Kit was used to quantify the endotoxin level of the purified particles, showing a value of 3.3 EU/mg, that is below the established threshold (35.7 EU/mg).

**Figure 3:**
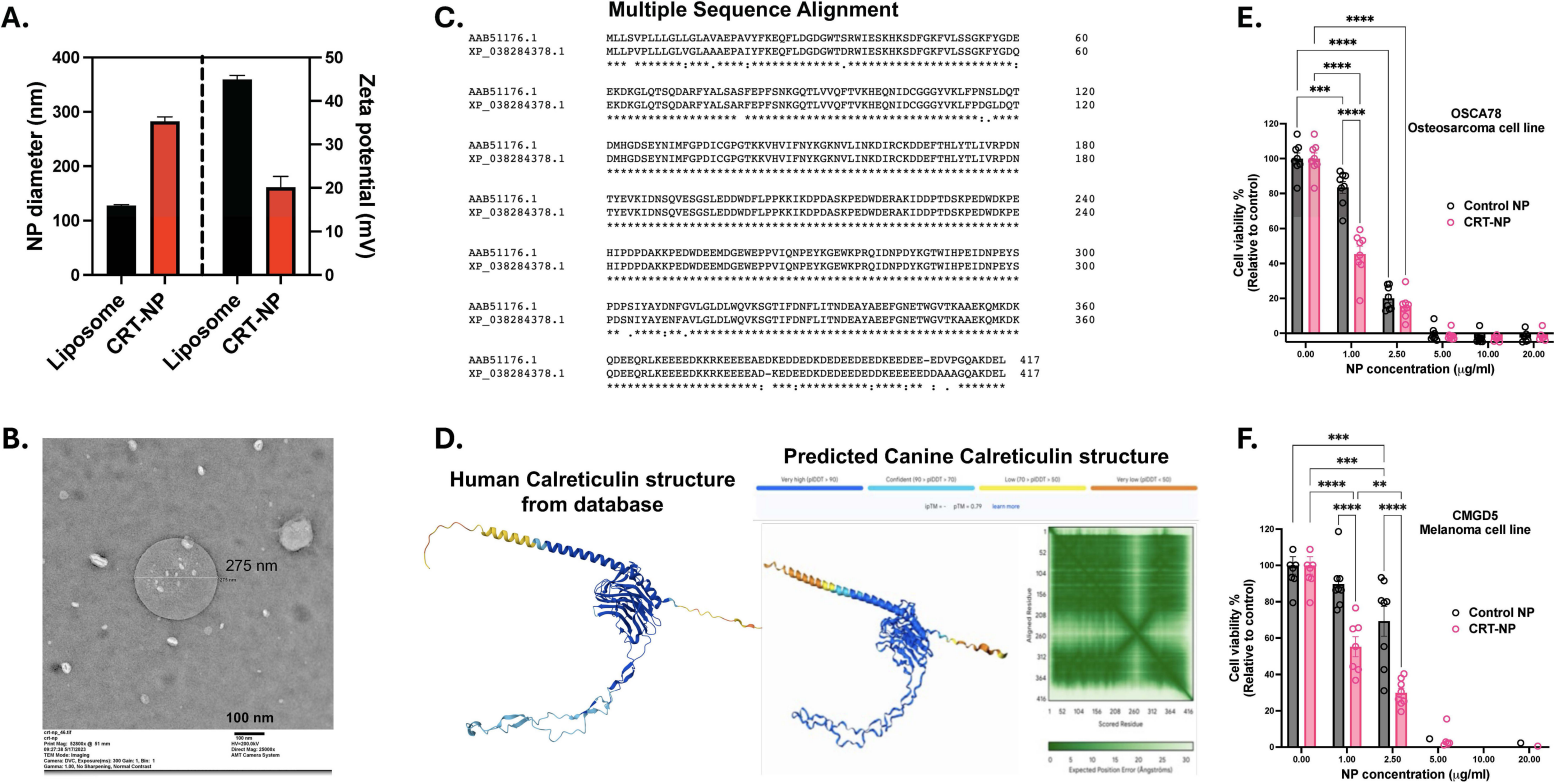
Characterization of CRT-NP. **(A)** Dynamic light scattering analysis for NP size indicating increase in hydrodynamic diameter from liposome to CRT-NP along with decrease in surface zeta potential changes indicating successful liposome-plasmid complex formation. **(B)** Transmission Electron microscopy (TEM) images showing morphological stability and similar size range of CRT-NP as observed in DLS analysis. **(C)** Multiple sequence alignment graph of human and dog CRT protein analyzed using Clustal Omega tool represented as (*) Identical conserved residue, (): Conservation between groups of strongly similar residue, (.) Conservation between groups of weakly similar residue. **(D)** Human CRT protein tertiary structure generated from EMBL-EBI-AlphaFold database. Dog CRT structure was predicted using Google Deepmind and Isomorphic labs structure prediction tool, AlphaFold3 with overall pTM score of 0.79. **(E, F)** MTT cytotoxicity graph of canine osteosarcoma cell line **(E)** and melanoma cell line **(F)** transfected with different concentration of CRT-NP. For Blank NP, liposomes were complexed with NTC plasmid with same plasmid backbone minus CRT gene. Statistical test: For cytotoxicity assay, Two Way ANOVA followed by Tukey test was employed. P value less than 0.05 considered significant for all tests. **p<0.005, ***p<0.0005, ****p<0.0001.

**Figure 4:**
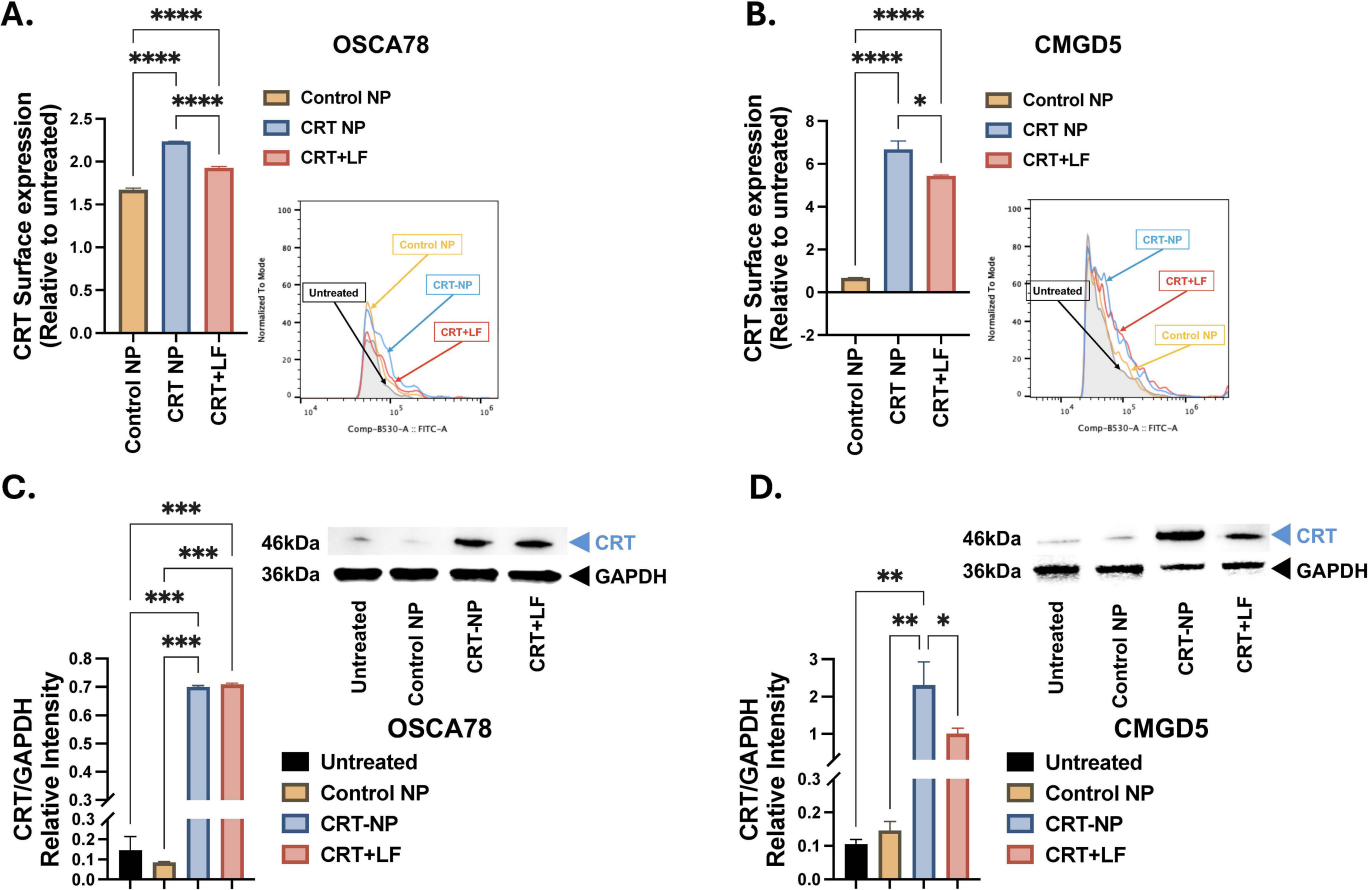
Evaluation of CRT expression in canine cell. **(A, B)** Surface expression of CRT protein on non-permeabilize canine cells was analyzed using flowcytometry 48h post-transfection with CRT-NP (1μg/ml CRT plasmid). Bar graphs representing foldchange in CRT surface expression relative to untreated OSCA78 osteosarcoma cell line **(A)** and CMGD5 melanoma cell line **(B)**. **(C, D)** Total CRT protein expression in CRT-NP (1μg/ml CRT plasmid) transfected cells was analyzed using western blot technique. Densitometry graph representing band intensity of CRT protein in OSCA78 **(C)** and CMGD5 **(D)** cell lines normalized using housekeeping protein, GAPDH, band intensity. For Blank NP, liposomes were complexed with NTC plasmid with same plasmid backbone minus CRT gene. CRT plasmid + Lipofectamine (1:3 ratio) complex was used as a positive control. Statistical test: One Way ANOVA followed by Tukey test was employed. P value less than 0.05 considered significant for all tests. *p<0.05, **p<0.005, ***p<0.0005, ****p<0.0001.

Cationic lysosomes upon incubation with CRT plasmid showed a shift in hydrodynamic diameter from 127.37 ± 1.78 nm to 282.39 ± 8.5 nm and surface charge (zeta potential) 44.94 ± 0.94 mV to 20.41 ± 2.49 mV (**Figure 3A**), indicating the formation of CRT plasmid-lipid complex, i.e. CRT-NP. PDI of CRT-NP was 0.164 ± 0.065. Stability and morphology of CRT-NP were confirmed using TEM which showed spherical CRT-NP with sizes within the range of DLS results (**Figure 3B**). Multiple sequence alignment of human and dog calreticulin protein showed 94.95% sequence similarity (**Figure 3C**) as well as tertiary structure of dog calreticulin predicted using AlphaFold showed high similarity to human calreticulin protein (**Figure 3D**). *In-vitro* cytotoxicity assay using canine cancer cell line showed that CRT-NP at 1μg/ml plasmid concentration (20μg/ml lipids) was more toxic than blank NP encapsulating NTC plasmid without CRT gene. Although at higher concentration, both CRT-NP and blank NP showed similar cytotoxicity due to increase in lipid content. Osteosarcoma cell line (**Figure 3E**) showed higher sensitivity for lipids in NP formulation compared to melanoma cell line (**Figure 3F**). Transfection of canine cell lines at non-lethal dose of NP (1μg/ml plasmid & 20μg/ml lipids), showed significant increase in CRT protein expression on cell surface of both OSCA78 (**Figure 4A**) and CMGD5 (**Figure 4B**) cells. OSCA78 showed ~2.2-fold increase whereas melanoma cell showed ~6.5-fold increase from control untreated cells. Expression of CRT protein in OSCA78 (**Figure 4C**) and CMGD5 (**Figure 4D**) cell lines was also confirmed by western blot.

### 3.2 CPMV and CRT-NP induced stable disease to partial remission in patients

**Figure 5:**
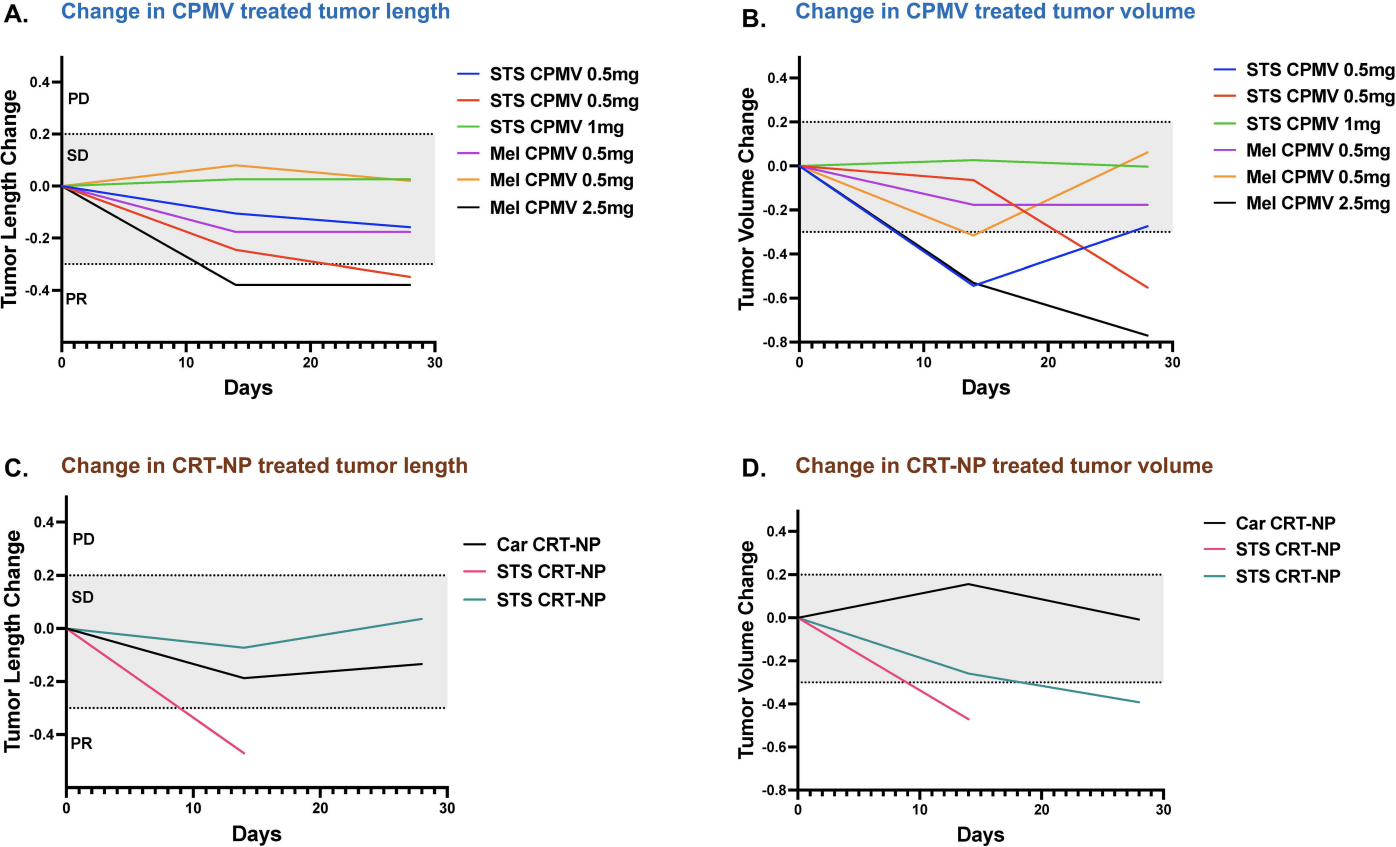
Therapeutic efficacy of CPMV and CRT-NP in canine cancer patients. **(A)** Swimmer plot showing changes in the longest tumor diameter (mm) for each patient treated with CPMV over 28 days post-treatment. 33% showed PR, while 66% showed SD. **(B)** Swimmer plot displaying changes in tumor volume (L*D²*0.5; mm³) for each patient treated with CPMV over the 28 days post-treatment period. 2 out of 6 patients showed a greater than 30% decrease in tumor volume. **(C)** Swimmer plot of CRT-NP treated patients showing changes in the longest tumor diameter (mm) over 28 days post-treatment. 2 out of 3 patients showed SD. **(D)** Swimmer plot displaying changes in tumor volume for each CRT-NP treated patient over the 28 days post-treatment initiation. 2 out of 3 patients showed a greater than 30% decrease in tumor volume. SD, Stable disease; PR, Partial Response; PD, Progressive Disease; STS, Soft Tissue Sarcoma; Car, Carcinoma; Mel, Melanoma.

CPMV induced stable disease (SD) in 4 out of 6 patients with melanoma and STS over the month-long treatment period (**Figure 5A**). One patient in each group achieved partial remission (PR). 2 out of 6 patients (33%) showed more than a 30% decrease in tumor volume by the 28th day of CPMV treatment (**Figure 5B**). Similarly, CRT-NP resulted in stable disease in patients with carcinoma (Car) and STS, with a second STS patient demonstrating partial remission before euthanasia (**Figure 5C**). Additionally, 2 out of 3 CRT-NP treated patients showed more than 30% decrease in tumor volume (**Figure 5D**). Summary of patient age, sex, disease type, and clinical response to nanoparticle treatment according to RECIST (V1.1) guidelines is shown in **Table 1**.

**Table 1:** Summary of patient age, sex, disease type, and clinical response to nanoparticle treatment according to RECIST (V1.1) guidelines.

BREED	GENDER	AGE	DIAGNOSIS	LOCATION	TREATMENT TYPE	NUMBER OF TREATMENT	TUMOR DIMENSIONS (mm)			RESPONSE
							PRE-TREATMENT	D14 POST	D28 POST	
LABRADOR RETRIEVER	FEMALE	8Y	CARCINOMA	MAMMARY GLANDS	CRT-NP	4	112*57	91*68	97*61	SD
PITTBULL MIX	FEMALE	11-12Y	SOFT TISSUE SARCOMA	LEFT FORE LIMB	CRT-NP	2	153*55	D7- 81*55; D11 euthanized		PR
PITTBULL BOXER MIX	MALE	10Y	SOFT TISSUE SARCOMA	LEFT HINDLIMB	CRT-NP	4	83*47	77*42	86*36	SD
LAB MIX	MALE	11Y	SOFT TISSUE SARCOMA	CHEST, LEFT LATERAL AREA	CPMV	4	19*14	17*10	16*13	SD
MINI SCHANAZEN	FEMALE	12-13Y	SOFT TISSUE SARCOMA	LEFT SHOULDER	CPMV	4	155*88	117*98	101*73	PR
LABRADOR	MALE	10Y	SOFT TISSUE SARCOMA	RIGHT FORE LIMB	CPMV	4	76*69	78*69	78*68	SD
AMERICAN BULLY	MALE	12Y	MELANOMA	ORAL	CPMV	4	51*21	42*21	42*21	SD
LABRADOR	MALE	9.5Y	MELANOMA	ORAL	CPMV	4	50*49	54*39	51*50	SD
RUSSELL MIX	MALE	13Y	MELANOMA	ORAL	CPMV	4	58*23	36*20	36*14	PR

SD, Stable Disease; PR, Partial Response. D0, Pre-treatment levels before 1st treatment; D14, 14 days after 1st treatment; D28, 28 days after 1st treatment.

### 3.3 CPMV treatment induced an anti-tumor immune response following IIT

**Figure 6:**
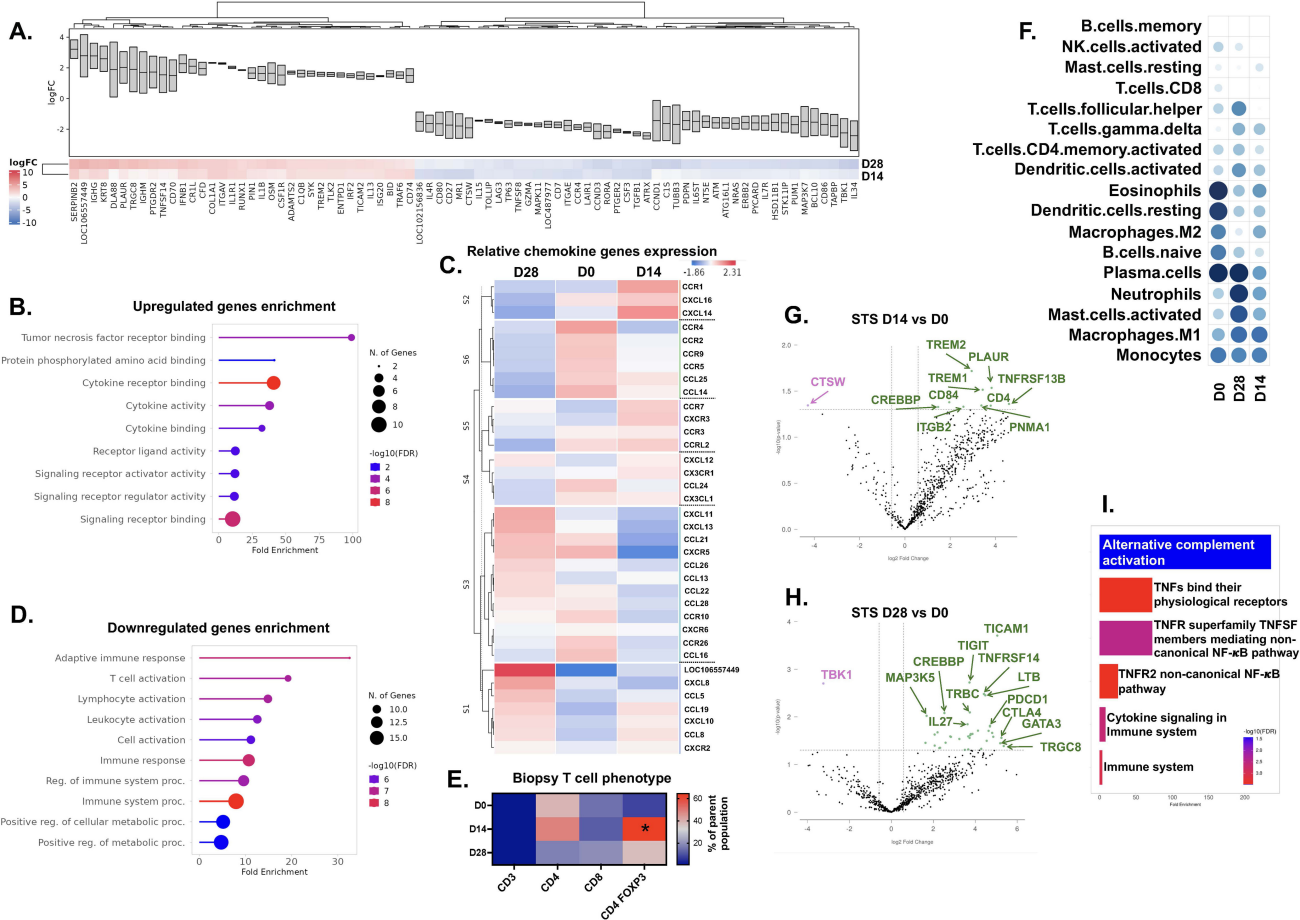
Nanostring Analysis of Immune Pathways with CPMV treatment in STS patients. **(A)** Common feature analysis of D14 & D28 STS biopsy samples on baseline of pretreatment samples (D0) represented as signature cluster plot showing expression foldchange (Log2) boxgraph and heatmap. **(B)** Gene Ontology (GO) enrichment analysis of upregulated genes in STS TME common to D14 & D28 samples on baseline of D0 samples. The x-axis shows fold enrichment in specific pathways, with bar colors representing –log10 (FDR) values. **(C)** Clustered chemokine genes expression heatmap representing relative expression of chemokine genes in STS TME sorted by timespan, D0vsD14vsD28. BigOmincs inbuilt database used to clustered functional chemokines genes into 6 sets. **(D)** GO enrichment analysis of downregulated genes in STS TME common to D14 & D28 samples on baseline of D0 samples. **(E)** Heatmap representing frequencies of CD3+ T cells and its phenotype analyzed by flowcytometry of STS biopsies collected at different timepoints. Heatmap is color coded based on cell percentage of gated parent population. **(F)** Dot plot depicting changes in immune cell abundance over time in STS TME following CPMV treatment. Immune cell populations were analyzed using the LM22 database and the non-negative least-square (I-NNLS) method via the BigOmics tool. **(G, H)** Volcano plot illustrating differentially expressed **(D, E)** genes significantly altered by CPMV treatment in STS TME at D14 **(G)** and D28 **(H)**, using D0 as the baseline. Pink dots indicate downregulated genes, while green dots represent upregulated genes. **(I)** Gene Ontology (GO) enrichment analysis of upregulated DE genes in STS TME at D28 vs. D0. The x-axis shows fold enrichment in specific pathways, with bar colors representing –log10 (FDR) values. STS, Soft Tissue Sarcoma; D0, Pre-treatment levels before 1st treatment; D14, 14 days after 1st treatment; D28, 28 days after 1st treatment.

Immune gene profiling of STS and melanoma tumors following CPMV treatment revealed distinct immune signatures. Functionally close genes that showed notable change in expression in STS tumors post CPMV treatment on baseline of pre-treated tumor biopsy were grouped to identify common functional signatures with CPMV treatment at D14 and D28 post-treatment (**Figure 6A**). Commonly upregulated genes were associated with GO-Biological functions like TNF receptor binding, cytokine activity, signaling and receptor binding, etc. (**Figure 6B**). As it was not clear which set of cytokine or chemokines were affected by CPMV treatment with gene enrichment analysis, we investigated expressional changes in chemokines genes specially. We observed in pretreatment STS biopsies, 9 chemokines genes showed higher expression, CCR4, CCR2, CCR9, CCR5, CCL25, CCL14, CCL24, CCR10, CCR26, CCL16 (**Figure 6C**). STRING analysis of these chemokines revealed their association with inflammation, mononuclear cell migration, lymphocyte and leukocyte chemotaxis, myeloid leukocyte migration, etc. (**Supplementary Figure S1A**). At D14 chemokine genes like CCR1, CXCL14, CCR7, CXCR3, CXCL12, CX3CR1 were upregulated, and they showed association with regulation of leukocyte chemotaxis, mononuclear cell migration and calcium ion transport and signaling (**Supplementary Figure S1B**). By D28, genes for chemokines like CXCL10, CXCL11, CXCL13, CXCL12, CXCL8, CCL5, CCL19, CCL8, CCL26, CCL13 were upregulated, and they were associated with lymphocyte, neutrophils and leukocyte chemotaxis, humoral immune response, regulation of leukocyte migration and cellular calcium ion homeostasis (**Supplementary Figure S1C**). Interestingly, downregulated gene sets identified in common feature analysis of D14 & D28 biopsies vs D0 (**Figure 6A**), enriched into functions like adaptive immune response, T cell and leukocyte activation, etc. (**Figure 6D**). To confirm this, we looked at tumor infiltrating T cell profile using flow cytometry and observed CD4+ FOXP3+ Treg cells increased significantly (~9.5 folds) in D14 biopsy samples compared to pretreatment sample, i.e. D0 (**Figure 6E**). By D28 Treg population decreased by 3.2 folds but still remained higher than pretreatment samples. immune cell profiling based on gene expression revealed decreased eosinophils, resting dendritic cells, and plasma cells, but an increase in the M1 macrophage gene expression on days 14 and 28 (**Figure 6F**). By day 28, a prominent increase in neutrophils, activated mast cells, and M1 macrophages, along with a slight increase in follicular helper T cells and activated dendritic cells, was observed. Additional statistical analysis of differential gene expression profiles following CPMV treatments revealed the upregulation of 10 differentially expressed genes associated with antigen processing (e.g., TNFSF13B, CD4, TREM1, TREM2, CD84, and CTSW) and 2 genes associated with cell proliferation and metastasis (ITGB2 and CREBBP; **Figure 6G**). Compared to days 0 and 14, day 28 STS tumors showed a relatively higher upregulation (36 genes vs. 12), along with the downregulation of TBK1, which is associated with cytokine and chemokine signaling (**Figure 6H**). Day 28 upregulated genes clustered into pathways associated with TNFs and receptor signaling, especially NFκB pathways (**Figure 6I**). The top 5 upregulated genes—TRGC8, CTLA4, GATA3, TICAM, and CD70—were associated with lymphocyte function and NFκB signaling. A 3.5-4.5 log2 fold increase in interferon-gamma-stimulating genes TBX21 and LOC483397 was observed, along with the simultaneous upregulation of regulatory genes IRF8, CTLA4, and PDCD1 (PD1).

**Figure 7:**
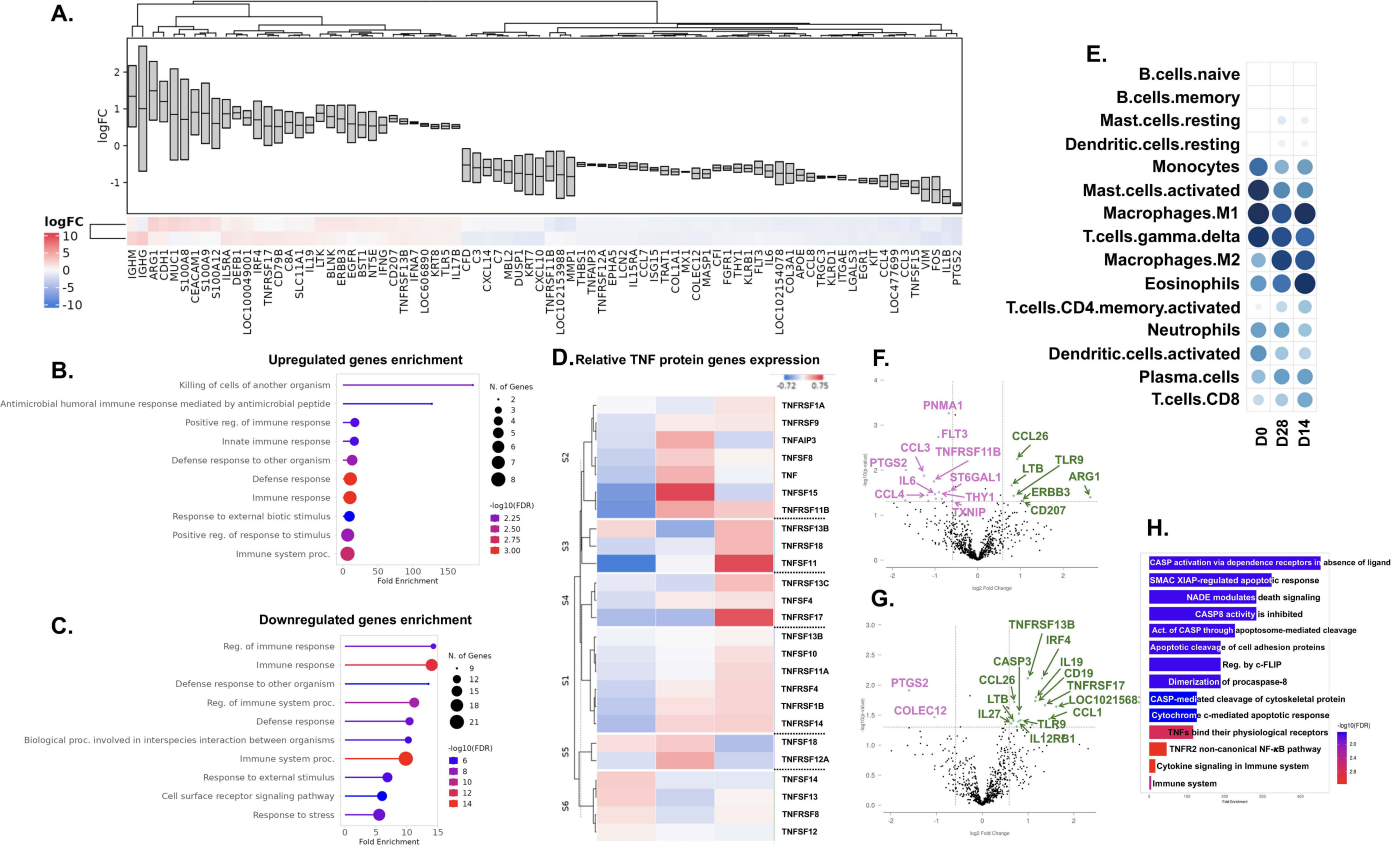
Nanostring Analysis of Immune Pathways with CPMV treatment in melanoma patients. **(A)** Common feature analysis of D14 & D28 melanoma biopsy samples on baseline of pretreatment samples (D0) represented as signature cluster plot showing expression foldchange (Log2) boxgraph and heatmap. **(B, C)** Gene Ontology (GO) enrichment analysis of upregulated genes **(B)** and downregulated genes **(C)** sets in melanoma TME common to D14 & D28 samples on baseline of D0 samples. The x-axis shows fold enrichment in specific pathways, with bar colors representing –log10 (FDR) values. **(D)** Clustered TNF protein family genes expression heatmap representing relative expression of TNF protein genes in melanoma TME sorted by timespan, D0vsD14vsD28. BigOmincs inbuilt database used to clustered functional TNF protein genes into 6 sets. **(E)** Dot plot depicting changes in immune cell abundance over time in melanoma TME following CPMV treatment. Immune cell populations were analyzed using the LM22 database and the non-negative least-square (I-NNLS) method via the BigOmics tool. **(F, G)** Volcano plot illustrating differentially expressed **(D, E)** genes significantly altered by CPMV treatment in melanoma TME at D14 **(F)** and D28 **(G)**, using D0 as the baseline. Pink dots indicate downregulated genes, while green dots represent upregulated genes. **(H)** Gene Ontology (GO) enrichment analysis of upregulated DE genes in melanoma TME at D28 vs. D0. The x-axis shows fold enrichment in specific pathways, with bar colors representing –log10 (FDR) values. D0, Pre-treatment levels before 1st treatment; D14, 14 days after 1st treatment; D28, 28 days after 1st treatment.

Upregulated genes identified by signature clustering of CPMV treated melanoma tumor samples (**Figure 7A**) clustered in several immune response associated pathway, but no distinct immune cell function was identified except for activation of humoral immune response post GO Biological functions analysis (**Figure 7B**). Similarly, downregulated genes also clustered into broader pathways like immune response, immune system processes and their regulations (**Figure 7C**). To identify which ‘biomarker’ was getting most affected with CPMV treatment in melanoma tumors, ranked-discriminant score for top features sets using BigOmincs inbuilt ‘meta’ method (p-value based) was computed. In D14 & D28 biopsy samples, genes associated with ‘TNF proteins’ feature set showed highest discrimination on the baseline of pretreatment melanoma samples (**Supplementary Figure S2A**). Relative expression profile of TNF protein family genes (**Figure 7D**) showed in pretreatment (D0) tumors, TNFRSF9, TNFAIP3, TNFSF8, TNF, TNFSF15, TNFRSF11B, TNFRSF12A, TNFSF18 genes had higher expression which upon STRING analysis showed association with pathways like regulation of leukocyte proliferation and activation, regulation of immune system processes and T cell proliferation, regulation of extrinsic apoptotic signaling pathway (**Supplementary Figure S2B**). In D14 samples, TNFSF14, TNFSF13, TNFRSF8, TNFSF12, TNFSF18, TNFRSF13B showed higher expression, and they were found to be associated with immune response and regulation of lymphocyte activation upon STRING analysis (**Supplementary Figure S2C**). On the other hand, D28 biopsy samples showed increased expression of TNFRSF13C, TNFSF4, TNFRSF17, TNFRSF13B, TNFRSF18, TNFSF11, TNFRSF1A, TNFRSF11B which are associated with pathways like TNF-mediated signaling pathway, regulation of B cell activation, positive regulation of T cell activation and inflammatory response, etc. (**Supplementary Figure S2D**). Immune cell profiling showed increased expression of M2 macrophages, eosinophils, activated memory CD4 T cells and CD8 T cells, with a decrease in monocytes, activated mast cells, and dendritic cells on days 14 and 28 post-treatment (**Figure 7E**). Statistical differential gene expression analysis showed initial CPMV treatment of melanoma tumors downregulated several protumoral genes, including IL1B, PTGS2, CCL3, CCL4, EGR1, IL6, FLT3, THY1 (CD90), SDC4, COLEC12, PNMA1, and TXNIP by day 14. Conversely, many genes associated with innate leukocytes, eosinophils, and myeloid cells were upregulated, such as TLR9, CCL26, CD207, ARG1, ERBB3, and LTB (**Figure 7F**). At the 28-day time point, many upregulated genes associated with death signaling and apoptotic response, primarily caspase-mediated apoptosis (**Figures 7G, H**). B cell-associated genes SPIB, CD19, IRF4, and TNFRS17 were also upregulated. The top upregulated gene, LOC102156836 (>1.6 log fold increase) associated with cytotoxic lymphocytes, while the next gene, CCL1 (1.46 log fold increase) associated with FOXP3+ Treg chemotaxis and stimulation. Similar to day 14, the expression of protumoral genes COLEC12 and PTGS2 decreased.

### 3.4 CPMV treatment induced increased activity of immune cells in blood and tumor tissues with distinct serum cytokine changes

**Figure 8:**
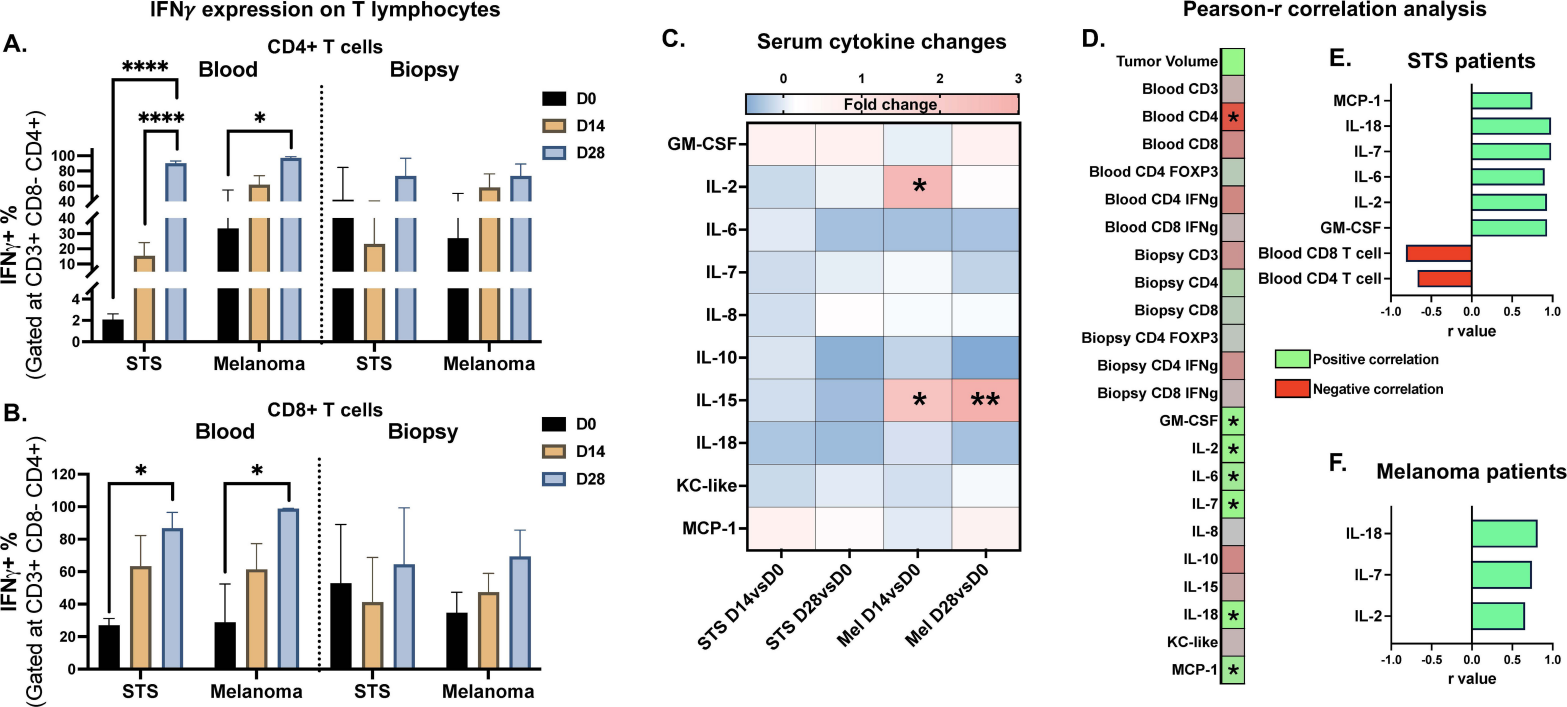
Immune analysis of blood and biopsies of CPMV treated patients. **(A, B)** Flow cytometric analysis of T cell populations in circulating blood and tumor infiltrates showed a significant increase in activated IFNγ+ CD4+ **(A)** and CD8+ T cells **(B)** in both the blood and tumor microenvironment following CPMV treatment in STS and melanoma patients. **(C)** Heatmap showing changes in serum cytokines over the baseline of initial levels (pre-treatment levels) on day 14 (D14vsD0) & day 28 (D28vsD0). CPMV stimulated different set of cytokines based on tumor type. **(D)** Heatmap showing Pearson-r correlation strength between immune signatures and tumor volume in CPMV treated patients irrespective of tumor type. **(E, F)** Significant Pearson correlations for tumor growth-immune signature (p<0.05) in CPMV treated STS **(E)** and melanoma **(F)** patients. X-axis representing the correlation coefficient and y-axis representing tumor volume-immune signature pairs. Statistical tests: Paired t-test was used to analyze changes in immune cell population and cytokine levels over time. Two-way ANOVA used further to analyze fold changes in cytokines levels among different tumor and treatment groups. Pearson-r analysis for correlation, negative correlation represented in red and positive correlation in green. Same color scheme used for correlation plots **(E, F)** P value less than 0.05 considered significant for all tests. *p<0.05, **p<0.005 for immune cell graph and cytokine changes. For Pearson-r correlation analysis all significant (p<0.05) associations irrespective of p-value strength are presented as *STS, Soft Tissue Sarcoma; Mel, Melanoma; D0, Pre-treatment levels before 1st treatment; D14, 14 days after 1st treatment; D28, 28 days after 1st treatment.

Blood and tumor samples were collected from 6/6 patients treated with CPMV. While the overall counts of CD3+, CD4+, and CD8+ T cells remained stable (data not shown), a marked increase in IFNγ expression was detected in circulating CD4+ cells (**Figure 8A**) and CD8+ T cells (**Figure 8B**) in patients with STS and melanoma 4 weeks after CPMV treatment. Like blood, biopsies tumors from these patients exhibited a similar upward trend in IFNγ expression in both CD4+ and CD8+ T cells with CPMV treatment. CPMV treatment induced a modest increase in GM-CSF and MCP-1 levels in STS and melanoma patients (**Figure 8C**). Additionally, IL-15 levels rose significantly up to 4 weeks post-CPMV treatment in melanoma patient. Unlike STS patients, melanoma patients exhibited a significant increase in serum IL-2 by D14, which then reverted to baseline by D28. Absolute serum levels of cytokines and chemokines are shown in **Supplementary Figure S3A** for STS patients and **Supplementary Figure S3B** for melanoma patients.

**Figure 9:**
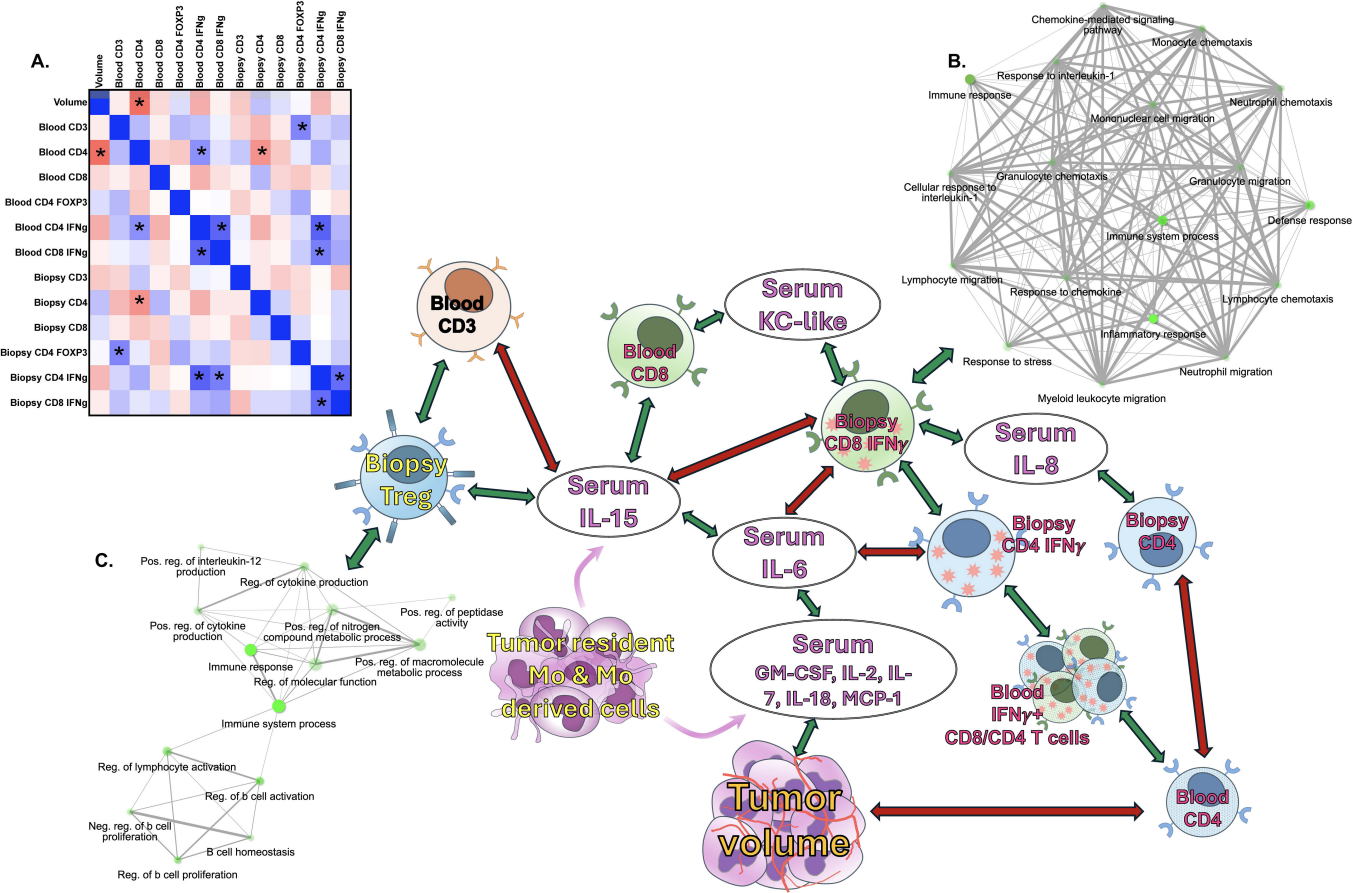
Correlation of immune activation with CPMV treatment. **(A)** Heatmap representing strength of Pearson-r correlation of tumor volume, immune cells and serum cytokines levels. Blue- positive correlation, Red-negative correlation. Asterisk boxes are significantly correlated variables (p<0.05). Summary of CPMV mediated immune activation in STS and melanoma patients was generated using significantly correlated variables including differential expression **(D, E)** genes identified from D28vsD0 STS samples and D28vsD0 melanoma samples analysis. Enrichment analysis of genes correlated with tumor infiltrating IFNγ+ CD8 T cells **(B)** and FOXP3+ CD4 Tregs **(C)** using canine STRING function database are represented as network graphs.

When analyzing the correlation between tumor volumes, serum cytokines/chemokines levels and immune cell populations in blood and biopsy samples, irrespective of tumor type, tumor progression with CPMV treatment positively correlated with serum levels of GM-CSF, IL-2, IL-6, IL-7, IL-18, and MCP-1, and negatively correlated with the abundance of circulating CD4+ T cells (**Figure 8D**). Tumor type segregation of correlation analysis showed STS tumor growth correlated significantly with increased serum levels of GM-CSF, IL-2, IL-6, IL-7, IL-18 & MCP-1, whereas increased circulatory CD4+ and CD8+ T cells numbers correlated with STS tumor regression with CPMV treatment (**Figure 8E**). On the hand, in melanoma patient no significant correlation with immune cell abundance was established but melanoma tumor growth showed significant correlation with higher serum levels of IL-18, IL-7 & IL-2 (**Figure 8F**). Correlation network (p<0.05 for Pearson-r correlation) of different immune signatures with tumor growth differentially expressed gene in TME with CPMV treatment irrespective of tumor type is shown in **Figure 9**.

### 3.5 CRT-NP induced anti-tumor immunity in canine patients

**Figure 10:**
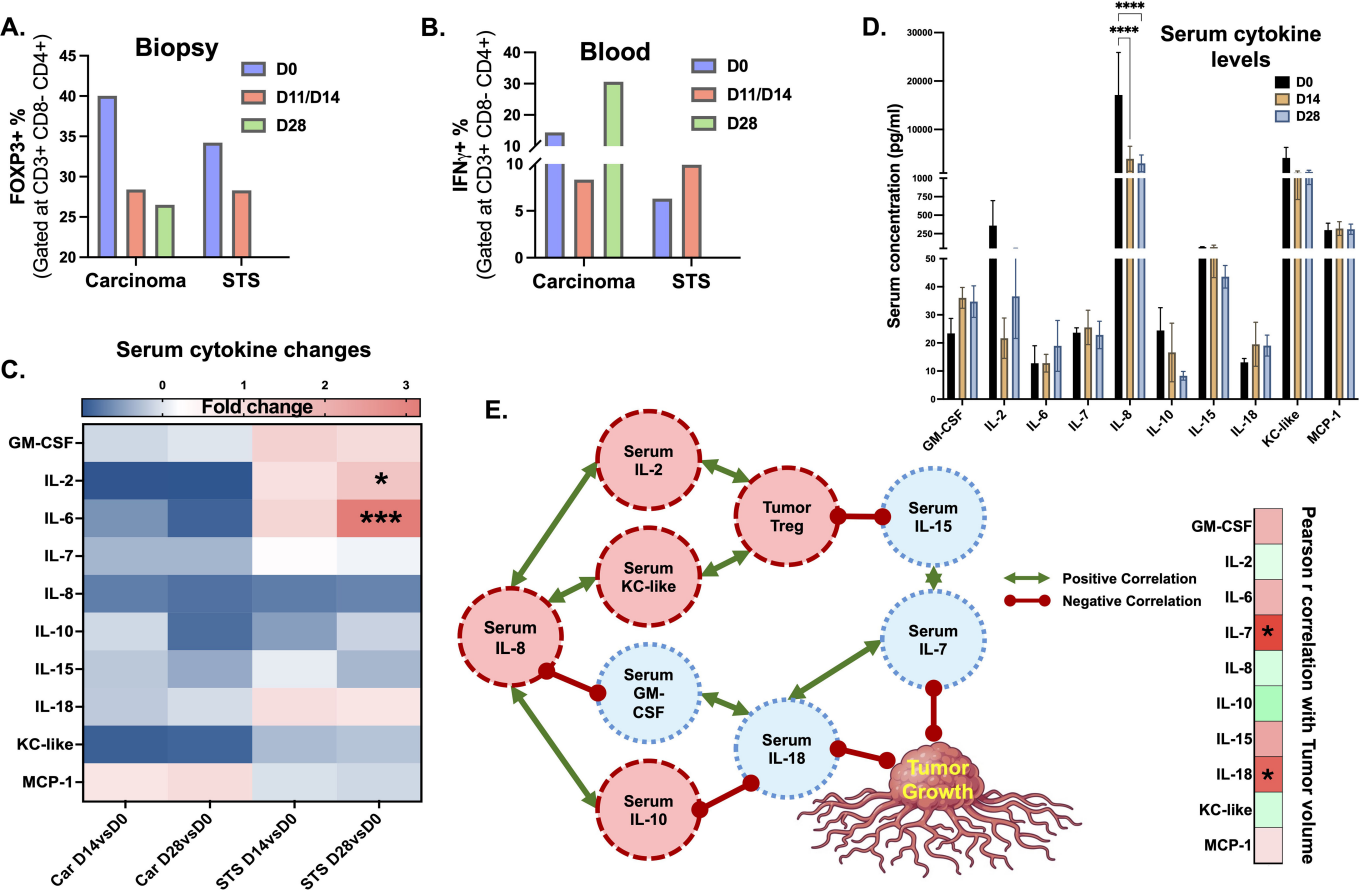
Immune analysis of blood and biopsies of CRT-NP treated patients. **(A, B)** Flow cytometric analysis of T cell populations circulating in blood and infiltrating tumor showed decrease in Treg (CD4+ FOXP3+) TME accumulation **(A)** and elevated activated IFNγ+ CD4+ **(B)** following CRT-NP treatment in carcinoma and STS patients. **(C)** Heatmap showing changes in serum cytokines over the baseline of initial levels (pre-treatment levels) on day 14 (D14vsD0) & day 28 (D28vsD0). **(D)** Bar graph showing absolute concentration of serum cytokine levels in CRT-NP treated patients irrespective of tumor type. **(E)** Correlation network of significantly correlated (p<0.05) immune signatures with IL-8 as center and tumor growth as outcome in CRT-NP treated patients. Positive correlation represented in green double head arrow, negative correlations as red dumbbell. Statistical tests: Paired t-test was used to analyze changes in immune cell population and cytokine levels over time. Two-way ANOVA used further to analyze fold changes in cytokines levels among different tumor and treatment groups. Two-way ANOVA was used to compare different cytokine concentrations (absolute) at different time point post CRT-NP treatment. Pearson-r analysis for correlation, negative correlation represented in red and positive correlation in green. Same color scheme used for correlation plot **(E)** P value less than 0.05 considered significant for all tests. *p<0.05, ***p<0.005, ****p<0.0001 for immune cell graph and cytokine changes. For Pearson-r correlation analysis all significant (p<0.05) associations irrespective of p-value strength are presented as *STS, Soft Tissue Sarcoma; Car, Carcinoma; D0, Pre-treatment levels before 1st treatment; D14: 14 days after 1st treatment; D28, 28 days after 1st treatment.

3 canine patients, 1 carcinoma (Car) and 2 STS, were treated with CRT-NP. Tumor samples were collected from 2/3 patients, 1 carcinoma and 1 STS patient. The STS patient was euthanized mid-study by the owners, and D0 & D11 comparisons (considered equivalent to day 14) were utilized for immune cell & cytokine analysis. Serum cytokine levels were compared for day 0,14, and 28 for 2/3 remaining patients (1 STS and 1 Car). With CRT-NP, decrease in tumor infiltration of Treg cells (CD4+ FOXP3+) cell was observed (**Figure 10A**) as well as increase in IFNγ expression on CD4+ T cells (**Figure 10B**). Immune gene analysis of carcinoma patient showed an increase in neutrophils, activated mast cells, and memory CD4 T cell signatures, along with a decrease in monocytes and follicular helper T cells 2 weeks post-treatment (**Supplementary Figure S4A**). In carcinoma patients, CRT-NP treatment enhanced MCP-1 serum levels whereas in STS patients GM-CSF, IL-2, IL-6 & IL-18 were enhanced (**Figure 10C**). Statistical test of serum cytokine levels of patients treated with CRT-NP irrespective of tumor type showed significant decrease in IL-8 levels (**Figure 10D**). Keeping IL-8 at center correlation network prepared with significant association showed that higher IL-8 levels were associated with higher levels of other pro-tumoral cytokines like IL-2, IL-10 and KC-like (**Figure 10E**). Whereas decreased IL-8 level was associated with increased dendritic cell stimulating GM-CSF levels. Higher IL-2 & KC-like levels indicated higher Treg accumulation in TME which was associated with reduced IL-15 levels. Pearson-r correlation between tumor volume and all immune signatures (**Supplementary Figure S4B**) revealed significant association of higher IL-7 and IL-18 with reduced tumor growth (**Figure 10E**).

## 4 Discussion

This pilot study in canine patients with spontaneous tumors highlights the potential of tumor immunomodulation through intratumoral administration of CPMV and CRT-NP. The results are encouraging, with 66% of patients achieving SD and 33% showing PR. These findings are consistent with previous reports on our eCPMV canine trials demonstrating significant melanoma and mammary tumor growth control with particle alone or in combination with radiation (27–29). This study is first to report therapeutic and immunomodulatory competence of CRT-NP in canine patients.

Our Nanostring findings align with previous preclinical research conducted in murine models, which demonstrated that CPMV effectively reprograms the TME from an immunosuppressive to an immunostimulatory state by activating innate immune cells, including M1 macrophages and neutrophils (5, 30). Nanostring evaluation of tumors on day 28 in STS patient treated with CPMV revealed a significant increase in gene expressions related to neutrophils, activated mast cells, and M1 macrophages, along with a modest rise in follicular helper T cells and activated dendritic cells. Neutrophils can adopt an antitumor phenotype in response to immunotherapy (31). Although the neutrophil response is often short-lived, some reports suggest that it can contribute to a sustained adaptive immune response and enhance the effectiveness of anti-PD1 therapy (32). This is partly achieved by creating an environment that promotes CD8+ T cell activation through the secretion of key activating factors, such as IL-12b. Similarly, M1 macrophages bolster the immune system’s ability to recognize and attack tumor cells by secreting various proinflammatory cytokines, including TNF-α, IL-12, and IL-6 (33). Although mast cells can exhibit both pro-tumoral and anti-tumoral effects, the observed changes in other cell types and cytokines within the STS TME suggest that they likely contributed to an antitumor phenotype in CPMV treated STS (34). In contrast, melanoma tumors treated with CPMV showed increased expression of M2 macrophages, eosinophils, and CD8 T cells, while there was a decrease in monocytes, activated mast cells, and dendritic cells on days 14 and 28 post-treatment. Eosinophils can enhance CD8 T cell recruitment and tumor cytotoxicity by promoting TNF-α and IFNγ signatures, potentially leading to prolonged overall survival, regardless of treatment type (35). Reduced dendritic cell signature could be associated with trafficking of DCs to the draining lymph node to present antigen to naïve T cells. Although detailed survival analysis was beyond the scope of this study, future research should explore immunomodulation factors in greater detail to correlate them with survival outcomes.

In our nanostring analysis, we observed distinct patterns of immunomodulation between STS and melanoma treated with CPMV. For STS, we found that the most prominently increased signaling pathway involved genes related to NFκB (Nuclear Factor kappa-light-chain-enhancer of activated B cells) signaling that is crucial for regulating immune responses, inflammation, and cell survival (36). In contrast, the most significantly upregulated genes in melanoma were associated with cytotoxic lymphocytes. Alongside this, there was a notable involvement of FOXP3+ Tregs (regulatory T cells) in melanoma. FOXP3+ Tregs are known for their role in maintaining immune tolerance and modulating immune responses, often dampening the activity of other immune cells (37). The stronger correlations in melanoma suggest a complex interplay between the recruitment of cytotoxic lymphocytes and the regulation by Tregs, potentially impacting the overall immune landscape within the TME. Despite the differential gene expression and signaling pathways observed, flow cytometry results showed that the overall abundance of tumor-infiltrating immune cells did not change significantly following intratumoral therapy. However, there was a notable increase in the proportion of IFNγ positive T cells in blood as well as in tumor biopsies. IFNγ is a critical cytokine produced by T cells that plays a vital role in enhancing the immune response against tumors, promoting anti-tumor activity, and influencing other immune cells’ functions (38). The increase in IFNγ positive T cells suggests that, although the total number of immune cells within the tumor did not fluctuate significantly, the functional state of these cells—particularly their ability to produce IFNγ was enhanced. IL-2 is crucial in the early stages of an anti-tumor T cell-dominant immune response, though persistently high levels could suppress CD8+ T cell activity by inducing exhaustion (39) or through the maintenance of Tregs. Conversely, IL-15, which is currently under investigation in clinical trials, stimulates anti-tumor immunity directly by promoting the proliferation and activation of CD8+ T cells and by activating NK/NKT cells (40, 41). This finding could imply that treatments were effectively stimulating a more aggressive CD8 T cell immune response, even if the overall cellular composition remains stable.

With CRT-NP, we observed an increase in serum MCP-1, suggesting heightened monocyte chemotaxis. However, cell profiling of carcinoma biopsies did not show a corresponding increase in monocytes or immunosuppressive M2 macrophages (**Supplementary Figure S4A**), in fact the monocytes signature decreased along with follicular helper cells. Instead, there was an increase in neutrophil populations and activated memory CD4 T cell signatures. Similarly, with flow cytometry analysis increase in circulatory activated CD4+ T cells (IFNγ+) and decrease in TME Tregs was observed with CRT-NP treatment (**Figures 10A, B**). MCP-1 can recruit neutrophils and promote NET formation (42, 43), so the elevated MCP-1 levels induced by treatment likely reflect enhanced neutrophil activation rather than increased monocyte chemotaxis or immunosuppression. Additionally, cytokine analysis in STS patients undergoing CRT-NP treatment revealed elevated levels of GM-CSF, IL-2, IL-6, and IL-18. Except for IL-2, GM-CSF, IL-6, and IL-18 showed a negative correlation with tumor growth in CRT-NP treated patients (**Figure 10E**; **Supplementary Figure S4B**). These cytokines have complex roles in anti-cancer immune responses. GM-CSF is crucial for macrophage activation and dendritic cell differentiation (44, 45). IL-6 has a dual role: intratumorally, it can suppress CD8+ T cell-mediated tumor cytotoxicity and promote tumor cell proliferation, whereas systemically, it is essential for T cell priming and migration (46–49). Despite not assessing IL-6 levels in CRT-NP treated tumors, the higher serum IL-6 levels and their negative correlation with tumor growth suggest an anti-tumor effect rather than a pro-tumoral one. Furthermore, sarcomas are known to be sensitive to NK cell activity, and IL-18 has been shown to enhance anti-tumor immunity in other NK cell-sensitive tumors, which may help explain our findings (50).

Our study has several limitations. The small sample size for both CPMV and CRT-NP restricted our ability to draw definitive conclusions regarding their applicability across diverse tumor subtypes and patient populations. Although early transcriptomic analysis of a carcinoma patient aligned with our previously published murine data, it was insufficient to fully characterize the immune outcomes induced by CRT-NP treatment. Future studies will be necessary to validate our findings more comprehensively. Additionally, further research is needed to assess immune response durability, resistance mechanisms, and the potential of combination therapies (e.g., checkpoint inhibitors) to enhance efficacy and survival with the NPs. Transitioning to clinical application will also require GMP production of NPs, which was beyond the scope of this study. Understanding how these NPs interact with the human tumor microenvironment particularly in antigen presentation, innate activation, and tumor evasion will be crucial for the development of phase 0 human trials.

In summary, while the immunomodulatory effects of NPs observed in this study including enhanced T-cell activation and correlations with cytokines such as IL-2, IL-6, and MCP-1 provide early mechanistic insights into key pathways, further investigation is needed to determine whether these outcomes are tumor-agnostic. In particular, enhanced CD8+ T-cell activation and reprogramming of the tumor microenvironment reflect mechanisms relevant to emerging immunotherapies in human oncology. Since most of our NP treatments resulted in stable disease in the patients, future studies would need to investigate the intratumoral NPs with other therapies (e.g., chemotherapy, radiation etc.) to improve outcomes. Additionally, optimizing dosage and frequency beyond the weekly regimen used in this study will be necessary to maximize therapeutic benefit. Lastly, while genomic tumor mapping is promising, establishing a reliable serum cytokine biomarker will be crucial for tracking treatment response to NPs.

## Data Availability

The original contributions presented in the study are included in the article/**Supplementary Material**. Further inquiries can be directed to the corresponding author.
