# Supplementary material for: Immunomodulatory effects of intratumoral cowpea mosaic virus and calreticulin nanoparticles in canine tumors: early results
Source: Front Immunol. 2025 May 2;16:1566394. doi: 10.3389/fimmu.2025.1566394 (PMC12082347; doi:10.3389/fimmu.2025.1566394)
Supplement: Supplementary file 1 [file DataSheet1.docx]

Supplementary Material

**Immunomodulatory Effects of Intratumoral Cowpea Mosaic Virus and Calreticulin Nanoparticles in Canine Tumors: Early Results**

Akansha Singh^1^, Jessica Fernanda Affonso de Oliveira^2,3,4^, Jessica Schrader^11^, Deepan Kishore^11^, Sri Vidhya Chandrasekar^1^, Steven Fiering^10^, Nicole F. Steinmetz^2-9^ and Ashish Ranjan^1*^

^1^Department of Radiation Oncology, UT Southwestern Medical Center, Dallas, TX

^2^Aiiso Yufeng Li Family Department of Chemical and Nanoengineering, University of California, San Diego, La Jolla, CA, United States

^3^Shu and K.C. Chien and Peter Farrell Collaboratory, University of California, San Diego, La Jolla, CA, USA

^4^Center for Nano-ImmunoEngineering, University of California, San Diego, La Jolla, CA, United States

^5^Department of Bioengineering, University of California, San Diego, La Jolla, CA, United States

^6^Department of Radiology, University of California, San Diego, La Jolla, CA, United States

^7^Institute for Materials Discovery and Design, University of California, San Diego, La Jolla, CA, United States

^8^Moores Cancer Center, University of California, San Diego, La Jolla, CA, United States

^9^Center for Engineering in Cancer, Institute of Engineering Medicine, University of California, San Diego, La Jolla, CA, United States

^10^Department of Microbiology and Immunology, Geisel School of Medicine at Dartmouth, Lebanon, New Hampshire

^11^Neel Veterinary Hospital, Oklahoma City

**Supplementary Figures:**


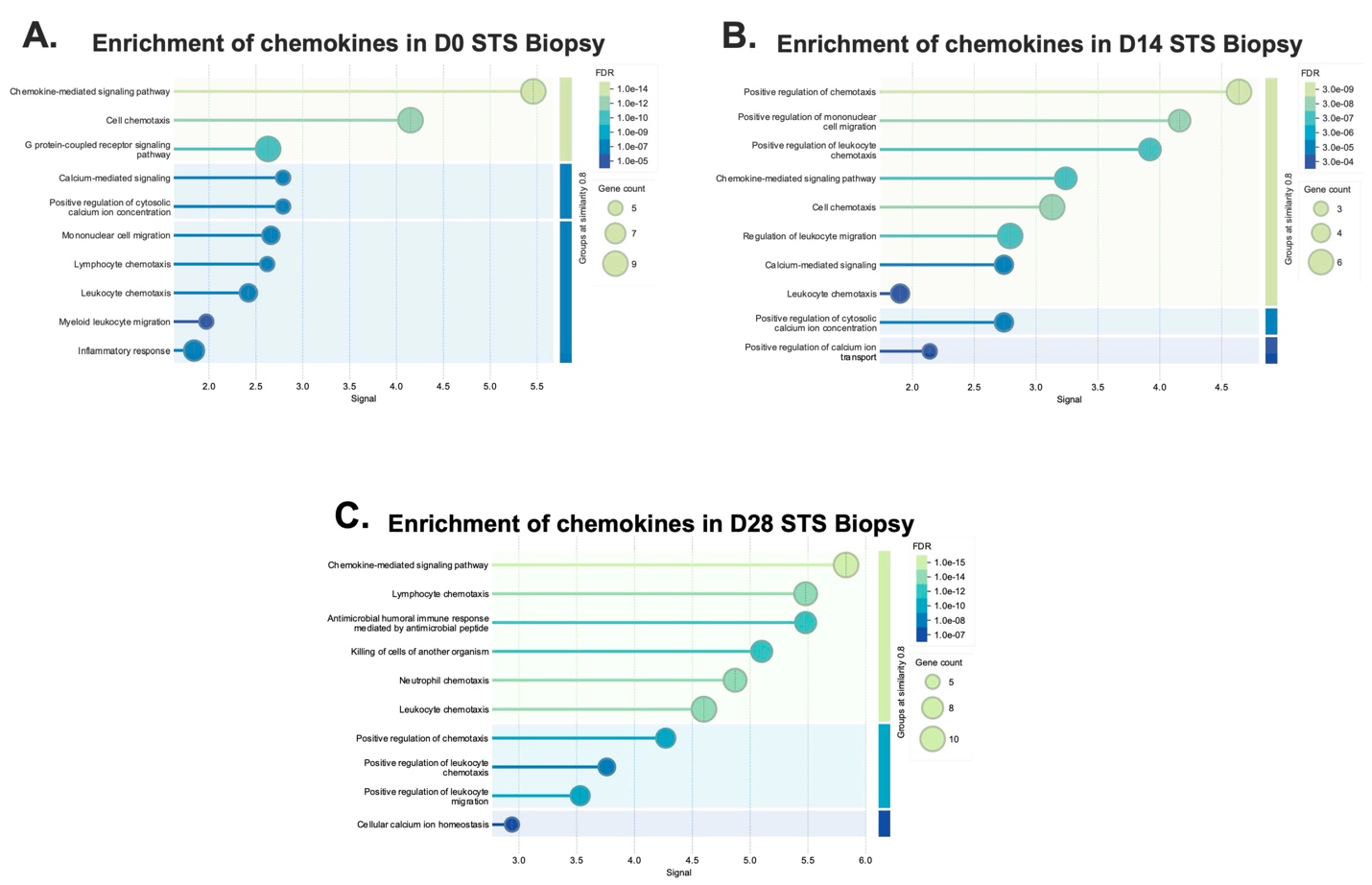


**Figure S1. STRING analysis of multiple proteins.** Differentially expressed chemokine genes at different timepoint in STS tumors treated with CPMV were analyzed to identify functional relationship and enrichment using GO biological function database. **A)** D0, pretreatment tumor samples, **B)** D14, 14 days after 1st CPMV treatment, **C)** D28, 28 days after 1st CPMV treatment. STS: Soft Tissue Sarcoma.


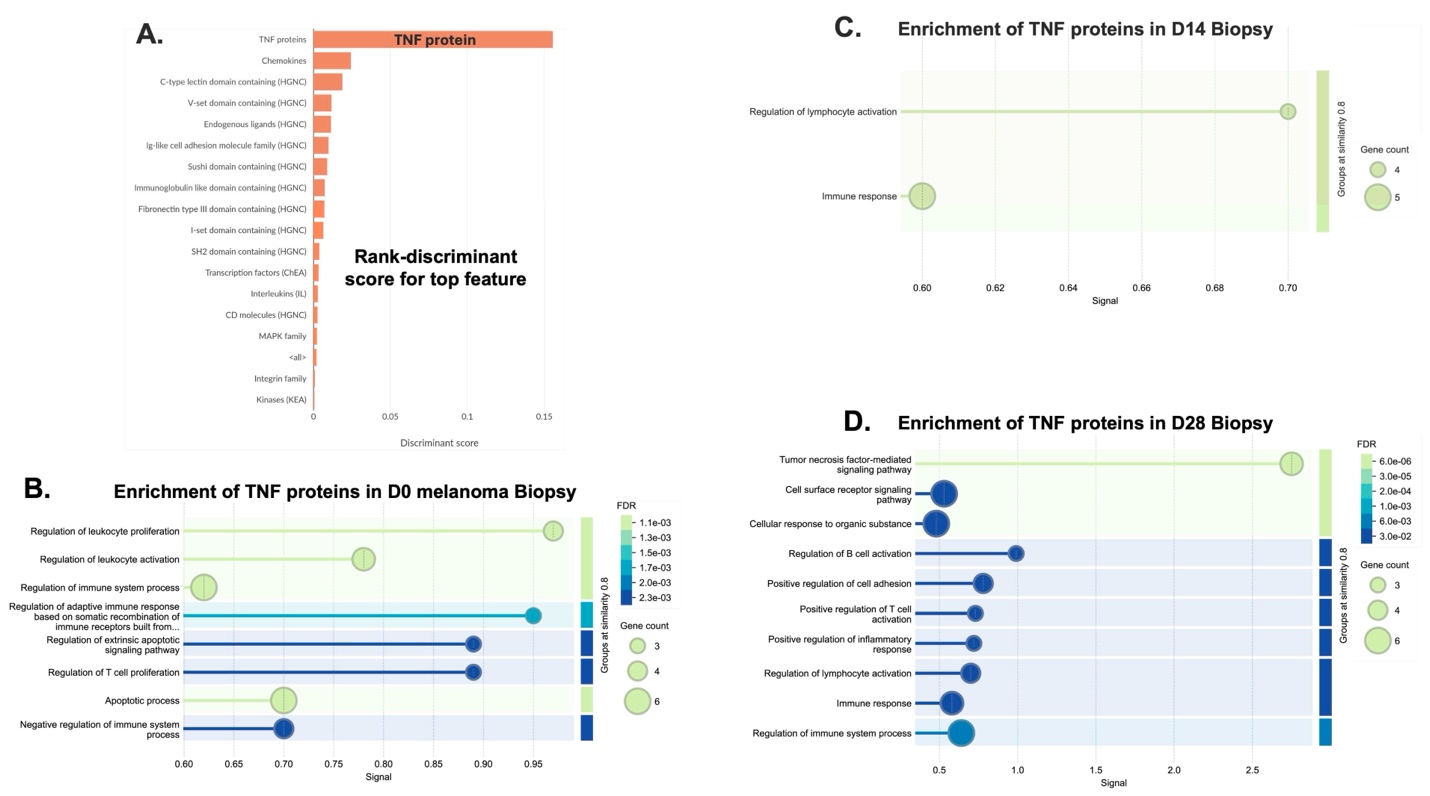


**Figure S2. A)** Rank-discriminant score graph prepared using ‘meta’ method (BigOmnics in-built test) representing most altered feature set based on gene expression changes in D14 & D28 melanoma biopsy samples post CPMV treatment on the baseline of pretreatment, D0, gene expression. **B-D)** Differentially expressed TNF protein genes at different timepoint in melanoma tumors treated with CPMV were analyzed to identify functional relationship and enrichment using GO biological function database. B) D0, pretreatment tumor samples, C) D14, 14 days after 1st CPMV treatment, D) D28, 28 days after 1st CPMV treatment.

**Figure S3. Serum cytokine levels (pg/ml) of CPMV treated patients. A&B)** Bar graph representing serum cytokines and chemokines levels of all (A) STS patients and (B) Melanoma patients at different timepoint irrespective of CPMV dose they received. STS: Soft Tissue Sarcoma; Mel: Melanoma; D0: Pre-treatment levels before 1st treatment; D14: 14 days after 1st treatment; D28: 28 days after 1st treatment.

**Figure S4. A)** Dot plot showing changes in immune cell abundance over time in carcinoma TME following CRT-NP treatment in two weeks post-treatment initiation. Immune cell populations were analyzed using the LM22 database and the non-negative least-square (I-NNLS) method via the BigOmics tool. **B)** Pearson-r correlation matrix between tumor growth and immune signatures in CRT-NP treated patients represented as heatmap. Heatmap is color coded based on r (correlation) value, negative correlation shown in red and positive correlation in green.
